# Disturbance‐Aware On‐Chip Training with Mitigation Schemes for Massively Parallel Computing in Analog Deep Learning Accelerator

**DOI:** 10.1002/advs.202417635

**Published:** 2025-05-20

**Authors:** Jaehyeon Kang, Jongun Won, Narae Han, Sangjun Hong, Jee‐Eun Yang, Sangwook Kim, Sangbum Kim

**Affiliations:** ^1^ Department of Material Science & Engineering Inter‐university Semiconductor Research Center (ISRC) Research Institute of Advanced Materials (RIAM) Seoul National University Seoul 08826 Republic of Korea; ^2^ Device Solutions Samsung Electronics Hwaseong 18479 Republic of Korea; ^3^ Samsung Advanced Institute of Technology (SAIT) Samsung Electronics Suwon‐si 16678 Republic of Korea

**Keywords:** Analog in‐memory computing, Disturbance, Disturbance‐aware training, Half‐selected, IGZO TFT, Neuromorphic, On‐chip training

## Abstract

On‐chip training in analog in‐memory computing (AIMC) holds great promise for reducing data latency and enabling user‐specific learning. However, analog synaptic devices face significant challenges, particularly during parallel weight updates in crossbar arrays, where non‐uniform programming and disturbances often arise. Despite their importance, the disturbances that occur during training are difficult to quantify based on a clear mechanism, and as a result, their impact on training performance remains underexplored. This work precisely identifies and quantifies the disturbance effects in 6T1C synaptic devices based on oxide semiconductors and capacitors, whose endurance and variation have been validated but encounter worsening disturbance effects with device scaling. By clarifying the disturbance mechanism, three simple operational schemes are proposed to mitigate these effects, with their efficacy validated through device array measurements. Furthermore, to evaluate learning feasibility in large‐scale arrays, real‐time disturbance‐aware training simulations are conducted by mapping synaptic arrays to convolutional neural networks for the CIFAR‐10 dataset. A software‐equivalent accuracy is achieved even under intensified disturbances, using a cell capacitor size of 50fF, comparable to dynamic random‐access memory. Combined with the inherent advantages of endurance and variation, this approach offers a practical solution for hardware‐based deep learning based on the 6T1C synaptic array.

## Graphical abstract

This study proposes novel operational schemes to solve the write disturbance issues in oxide‐semiconductor and capacitor‐based synaptic devices (6T1C devices). These schemes effectively neutralize disturbances, enabling high‐performance on‐chip training of convolutional neural networks and reducing capacitor size over 100 times. Combined with the advantages of the 6T1C device, this approach offers a practical solution for analog in‐memory computing by controlling unavoidable training disturbances.

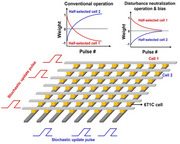

## 1 Introduction

Implementing neural networks in hardware offers the distinct advantage of parallel data processing with low power consumption, setting it apart from traditional computing systems.^[^ ^1^ ^]^ Hardware‐based neural networks can be categorized by their training method: off‐chip and on‐chip training. Off‐chip training, where weights are determined in software and transferred to hardware for inference, leverages Ohm's law and Kirchhoff's law to enable O(1) Multiply‐Accumulate operations, making it an effective approach for accelerating inference. Despite this advantage, off‐chip training is inherently limited by privacy concerns arising from the necessity of data transmission to the cloud, which may expose sensitive user information to potential security risks. Furthermore, the approach is vulnerable to hardware non‐idealities such as defective devices and wiring resistance, and it poses significant challenges in adapting to user‐specific data in real‐time.^[^ ^2^, ^3^ ^]^ These limitations can undermine the benefits of hardware implementations.

On‐chip training, on the other hand, allows hardware to compensate for imperfections during training,^[^ ^4^, ^5^ ^]^ eliminating the hardware implementation drawback of off‐chip training while offering advantages in terms of user data security. As the training phase requires significantly higher computations than inference, hardware implementation offers greater benefits at this stage. However, this approach faces significant challenges due to the lack of suitable synaptic devices. These devices must offer linearity and symmetry in weight updates, high endurance to withstand numerous update pulses, and uniformity, retention, and compact cell area.^[^ ^6^ ^]^ While achieving linearity and symmetry has traditionally been the major hurdle in hardware‐based deep learning, recent advances in training algorithms that relax these stringent requirements have shifted the focus to intrinsic device properties such as endurance, uniformity, and retention.^[^ ^7^, ^8^, ^9^, ^10^ ^]^

**Figure 1:**
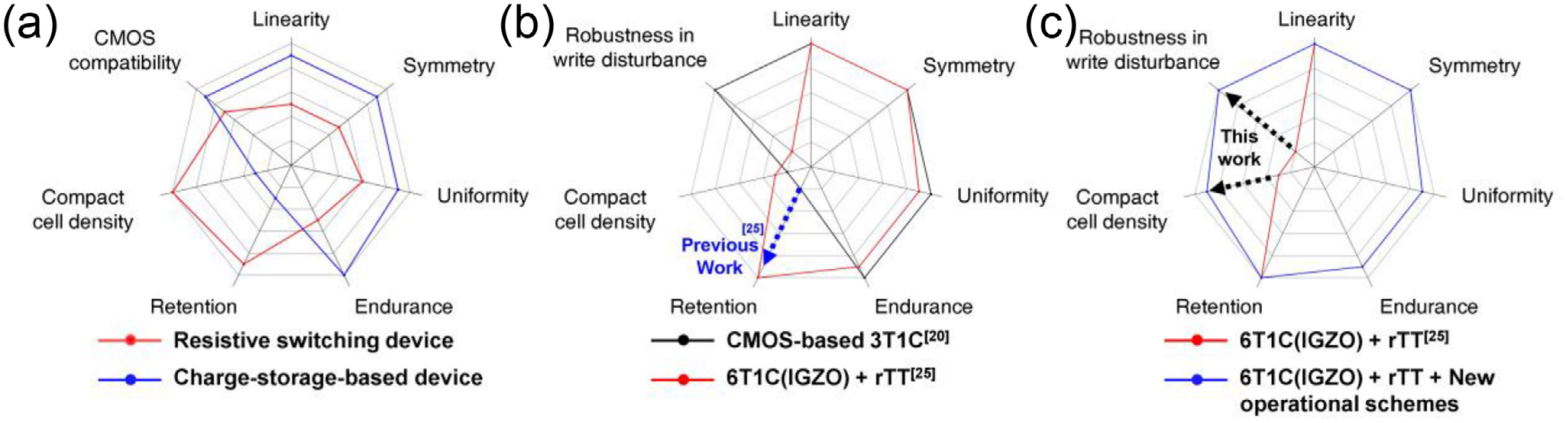
a) Spider chart illustrating the key characteristics required for synaptic devices in neural network training, along with the advantages and disadvantages of the resistive switching devices and charge‐storage‐based devices. Attributes further from the center indicate greater advantages in the respective categories. b) Comparison of the advantages and disadvantages of Si‐CMOS 3T1C and 6T1C + rTT. The integration of IGZO, which has a low leakage current, with the rTT algorithm successfully resolves the retention issues that could not be addressed in 3T1C devices. c) Comparison between the previous work based on 6T1C + rTT and this work based on 6T1C + rTT + new operational methods. This work successfully addresses the half‐selected issue in the crossbar array and the large cell area problem caused by the capacitor, which remained unresolved in the previous work.

Resistive switching devices, like phase‐change memory^[^ ^11^ ^]^ and resistive random‐access memory (RRAM),^[^ ^12^, ^13^, ^14^, ^15^ ^]^ are studied for on‐chip training due to their strong retention and compact cell size. Moreover, the integration of RRAM with training algorithms designed to effectively mitigate its inherent device‐level limitations – such as insufficient weight update linearity and symmetry, a limited number of multi‐level states, and noise during the conductance readout process – have demonstrated that a well‐designed training algorithm can compensate for these non‐idealities, demonstrating the feasibility of hardware‐based training.^[^ ^10^, ^16^ ^]^ However, the atomic‐level conductance modulation mechanism of resistive switching devices exhibits poor uniformity and endurance,^[^ ^17^ ^]^ particularly lacking sufficient endurance for numerous update pulses.^[^ ^18^, ^19^ ^]^ This endurance limitation cannot be fully mitigated through algorithmic compensation, imposing fundamental constraints on the hardware implementation (**Figure** **1**a). (Details in S1, Supporting Information)

Charge‐storage‐based synaptic devices, using well‐established components such as capacitors, complementary metal oxide semiconductors (CMOS), and oxide semiconductors, demonstrate superior CMOS compatibility and endurance^[^ ^20^, ^21^ ^]^ key challenges that remain difficult to overcome even with algorithmic optimizations. (Figure 1a). However, these devices face issues such as poor retention and large cell size due to their dependence on capacitors.^[^ ^20^, ^22^, ^23^ ^]^ Specifically, Si CMOS‐based synaptic devices suffer from poor retention issues due to the high leakage currents inherent in CMOS. Despite these challenges, capacitor‐based devices remain attractive for on‐chip training, driving efforts to address their limitations.

One promising approach involves integrating InGaZnO (IGZO) Thin‐Film Transistors (TFTs) with capacitors to replace CMOS, as the low leakage currents of IGZO TFTs significantly improve retention.^[^ ^24^, ^25^ ^]^ Notably, a recent breakthrough, the IGZO TFT‐based 6T1C device, due to its innovative structure, demonstrated linearity and symmetry using only IGZO n‐channel FETs. Furthermore, when paired with the retention‐centric Tiki‐Taka (rTT) algorithm, this device effectively addresses retention issues, making it a strong candidate for on‐chip training (Figure 1b).^[^ ^25^ ^]^

Although 6T1C devices have recently shown promise in simple on‐chip training tasks based on their excellent characteristics,^[^ ^25^, ^26^ ^]^ several challenges remain in practice. First, the 6T1C faces challenges related to significant write disturbance issues in the half‐selected (HS) conditions. To enable fully parallel weight updates, a stochastic update scheme is used, with stochastic update pulses applied along the row and column directions, corresponding to input data and gradients, respectively.^[^ ^27^ ^]^ In the 6T1C devices, weight updates occur only when the two transistors responsible for updates are simultaneously selected. However, the stochastic update scheme does not always select both update transistors, resulting in frequent HS cases. The write disturbance issue in HS cases^[^ ^28^ ^]^ is particularly severe in 6T1C, as the floating capacitor nodes make the device more susceptible to parasitic capacitance within the device, leading to numerous unintended effects in HS cases. Moreover, the 6T1C's susceptibility to parasitic capacitance necessitates a larger capacitor, increasing the cell area. As the capacitor size is reduced, the impact of parasitic capacitance intensifies, ultimately leading to an overall increase in chip size. (Figure 1b)

Here, we devised three simple and improved operational schemes that effectively resolve the remaining challenges of 6T1C. These methods, developed through in‐depth analysis of 6T1C operation and the rTT algorithm, involve the intentional application of pulses to specific lines during the weight update phase, the utilization of optimized bias conditions, and the scheduling of update pulse sequences. Through device‐algorithm co‐optimization, these schemes successfully mitigate accuracy degradation caused by write disturbances stemming from HS cases (Figure 1c). First, we demonstrated that the proposed operational methods can effectively alleviate HS issues in both a single 6T1C cell and a crossbar array. Second, despite accurately reflecting all write disturbances caused by HS cases in real‐time during training, we demonstrated that these methods achieved software‐equivalent learning accuracy of ≈93% on the CIFAR‐10 dataset, even in convolutional neural networks (CNN), where write disturbances are exacerbated by numerous update pulses. Notably, this study is the first to precisely account for all write disturbances in HS cells, which are inevitably involved in fully parallel updates. Lastly, these methods pave the way to reduce the size of the cell capacitor to 50fF, comparable to that of dynamic random‐access memory (DRAM),^[^ ^29^ ^]^ thereby solving the cell area problem (Figure 1c). Despite effectively addressing the challenges of 6T1C, these proposed schemes neither increase the complexity of peripheral circuits nor extend the required training time, making them highly efficient to implement.

## 2 Results and Discussion

### 2.1 Unveiling and Mitigating Disturbance Effects in Synaptic Devices

**Figure 2:**
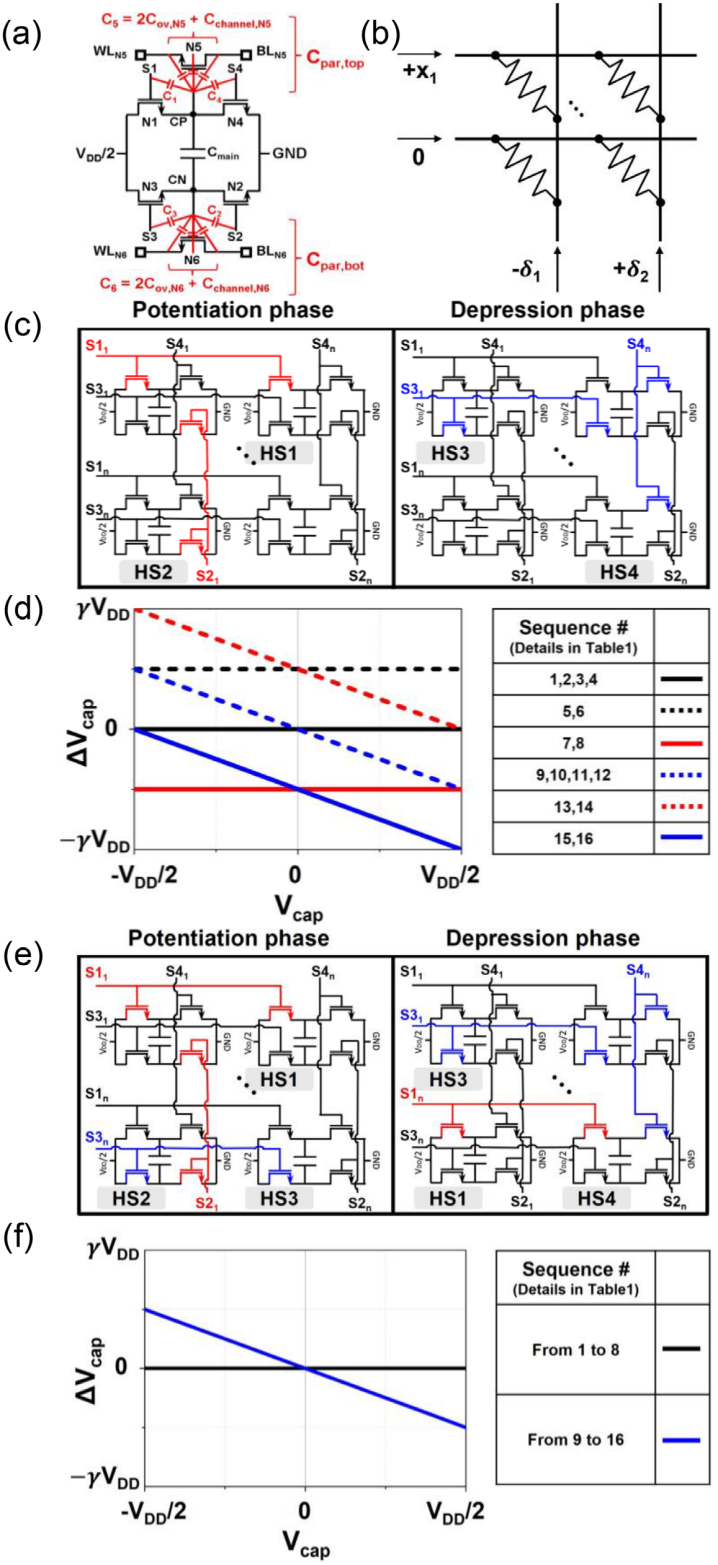
a) Schematic of 6T1C single cell including parasitic capacitors b) Update scenario in a 6T1C crossbar array c) Update process performed under conventional operation based on the update scenario in (b). d) Disturbance trends induced by HS sequences under conventional operation e) Update process performed under DNO based on the update scenario in (b). f) Disturbance trends induced by HS sequences under DNO.

During deep learning training in a crossbar array, the synaptic cell at (i,j) updates its weight according to Equation (1):

(1) $$\Delta w_{ij}=-\eta x_{i}\delta_{j}$$

where η is the learning rate, and the values of *x_i_*, δ_*j*_ are obtained through feedforward and backpropagation, respectively. In the 6T1C (**Figure** **2**a), the pulse generation probability for *x* in Equation (1) is applied to S1 (S3) along the row direction, while the pulse generation probability for δ is applied to S2 (S4) along the column direction. Weight updates occur only when both pulses coincide, with potentiation when N1 and N2 are simultaneously selected and depression when N3 and N4 are simultaneously selected. However, during the stochastic pulse application process, cases frequently arise where only one transistor from N1 to N4 is selected. Ideally, weight change should not occur in these cases. However, in the 6T1C, due to its floating capacitor terminals, both CP and CN nodes in Figure 2a change simultaneously when a single transistor is selected. This causes charge distribution between the main and parasitic capacitors within the device (Figure 2a), leading to weight changes.

When one transistor is selected in the 6T1C, the disturbance from charge distribution is described by Equations (2) and (3). Equation (2) corresponds to the case where the transistor connected to the CP node (N1 or N4) is selected, while Equation (3) addresses the case for the transistor connected to the CN node (N2 or N3).

(2) $$\Delta V_{cap,disturb}=\frac{C_{par,bot}}{C_{main}+C_{par,bot}}\left(V_{CP,final}-V_{CP,initial}\right)$$

(3) $$\Delta V_{cap,disturb}=-\frac{C_{par,top}}{C_{main}+C_{par,top}}\left(V_{CN,final}-V_{CN,initial}\right)$$

In Equations (2) and (3), C_main_ represents the capacitance of the main capacitor in the 6T1C cell, while C_par,top_ and C_par,bot_ denote the parasitic capacitance present at the CP and CN nodes, respectively, as shown in Figure 2a. V_cap_ is defined as V_CP_ – V_CN_, V_initial_ and V_final_ represent the voltage before and after the transistor selection, respectively. In Equations (2) and (3), the values of V_CP,final_, and V_CN,final_ depend on the transistor currently selected. For example, in Equation (2), V_CP,final_ is set to V_DD_/2 for the N1 selection and to GND for an N4 selection. Similarly, V_CP,initial_, and V_CN,initial_ depend on the previously selected transistor and the charge stored in the capacitor (V_cap_).

To thoroughly analyze the disturbance, it is essential to evaluate them considering the sequence of selected transistors across the full capacitor voltage range (from ‐V_DD_/2 to V_DD_/2). Under conventional operation (Figure 2c), excluding cases where pulses coincide and updates occur, there are four possible HS cases for transistors (from N1 to N4). These cases are designated as HS1, HS2, HS3, and HS4, as depicted in Figure 2c. These cases result in 16 possible sequences (**Table** **1**). Figure 2d shows the disturbance trends induced by the subsequent case in the sequences listed in Table 1, based on Equations (2) and (3). (Details provided in S2, Supporting Information). Note that while C_par,top_ and C_par,bot_ vary with bias conditions but they were assumed constant for simplicity in the plot, and the γ shown in the plot are defined from Equation (4).

**Table 1:** The 16 transistor selection sequences and their corresponding sequence numbers.

Number	Sequence	Number	Sequence	Number	Sequence	Number	Sequence
1	HS1 → HS1	5	HS4 → HS1	9	HS1 → HS3	13	HS1 → HS2
2	HS2 → HS2	6	HS3 → HS2	10	HS3 → HS1	14	HS2 → HS1
3	HS3 → HS3	7	HS1 → HS4	11	HS2 → HS4	15	HS3 → HS4
4	HS4 → HS4	8	HS2 → HS3	12	HS4 → HS2	16	HS4 → HS3

As shown in Figure 2d, the disturbance behavior varies with the sequence. First, when the same HS case occurs successively, V_initial_ and V_final_ for the subsequent case remain the same, resulting in no disturbance. Second, when different transistors on the same C_main_ node (CP or CN) are selected successively, the magnitude of disturbance remains constant regardless of V_cap_. Third, when transistors connected to different C_main_ nodes are selected alternately, the magnitude of disturbance varies depending on V_cap_. During training, stochastically applied pulses and varying V_cap_ across cells make it impossible to predict disturbances, resulting in the continuous recording of inaccurate information onto the device and severely impairing training performance.^[^ ^8^, ^9^, ^10^ ^]^

Therefore, we focused on developing solutions to mitigate and manage the effects of these random disturbances. An improved operational scheme, as illustrated in Figure 2e was devised, which does not require additional complexity in peripheral circuitry. This approach has been termed the disturbance neutralization operation (DNO).

In the conventional potentiation (depression) phase, a stochastic pulse for *x* in Equation (1) is applied to the S1 (S3) line, while the stochastic pulse for δ is applied to the S2 (S4) line. However, in the DNO, during the potentiation (depression) phase, all non‐activated rows for S1 (S3) (e.g., the nth row line in Figure 2e) are intentionally activated by applying S3 (S1), ensuring that the N1 or N3 transistor is always activated. Figure 2e shows the four HS cases that can occur in DNO. Note that while HS2 and HS4 share the same names as those in the conventional operation, the transistors selected in these cases are different.

Figure 2f illustrates the disturbance trends caused by the 16 sequences (Table 1) in DNO. As shown, the DNO effectively suppresses disturbances in sequences where transistors connected to the same node are selected. (Black line in Figure 2f). This behavior stems from the differences in pulse widths among the transistors in the 6T1C. The extent of disturbance is determined by V_initial_ and V_final_, as described in Equations (2) and (3). In the 6T1C, the pulse width of S1 (S3) is longer than S2 (S4), causing N1 (N3) to turn off later, making it the primary transistor that determines V_final_. Consequently, regardless of whether N2 (N4) is selected, the sequence is equivalent to the repetition of the same HS cases, resulting in no disturbance.

Under the DNO, weight disturbance occurs only in sequences where transistors connected to different nodes (CP and CN) are selected alternately. (Blue line in Figure 2f). In this case, with N1 or N3 consistently activated, the CP or CN node remains at V_DD_/2, and the magnitude of the disturbance in such sequences can be constrained by Equation (5), where the parameter γ is defined in Equation (4). (Details in S2, Supporting Information).

(4) $$\gamma =\frac{C_{par,top}}{C_{main}+C_{par,top}}=\frac{C_{par,bot}}{C_{main}+C_{par,bot}}$$

(5) $$\Delta V_{cap,disturb}=-\gamma V_{cap}$$

The DNO not only reduces the number of disturbance sequence cases but also confines disturbances to a decay‐like behavior toward V_cap_ = 0. Note that the potentiation or depression does not affect the decay‐like disturbance behavior, as one of the CP or CN nodes remains fixed at V_DD_/2. Although such decay‐like disturbances persist under the DNO, prior studies demonstrate that co‐optimizing the device (6T1C) and algorithm (rTT) effectively mitigates such decay from retention issues, leveraging them as regularization to enhance performance.^[^ ^25^ ^]^ Therefore, operating the 6T1C under the DNO is expected to significantly mitigate disturbance effects.

**Figure 3:**
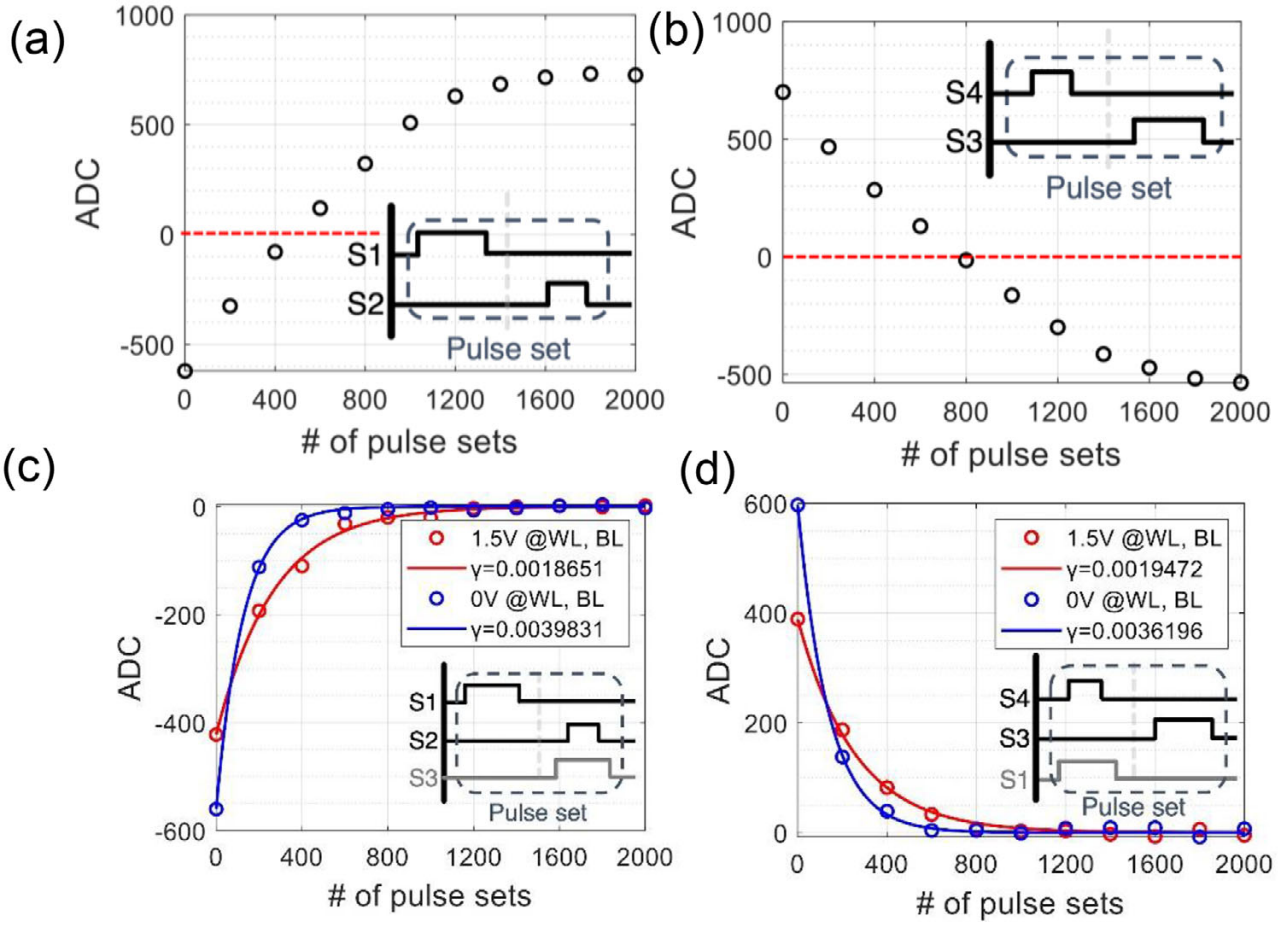
a) ADC changes observed during the repeated application of sequence 13,14 under conventional operation. The red dashed line represents the ADC when V_cap_ = 0. b) ADC changes observed during the repeated application of sequence 16,15 under conventional operation (c) ADC changes observed during the repeated application of sequence 13,14 under DNO (d) ADC changes observed during the repeated application of sequence 16,15 under DNO. In (c) and (d), the ADC with V_cap_ = 0 corresponds to 0.

We validated the DNO through device measurements under the experimental conditions most affected by disturbance. The measurements in 6T1C were conducted entirely using a printed circuit board (PCB) based on a discrete device and microcontroller unit (MCU), which was fabricated in prior studies^[^ ^25^ ^]^ (See the Experimental Section). **Figure** **3**a,b shows that, under conventional operation, repeated sequence numbers 13,14 and 16,15 from Table 1 lead to convergence at V_DD_/2, ‐V_DD_/2, as described in Figure 2d. However, with the DNO, repeated sequence numbers 13,14 and 16,15 exhibited decay‐like disturbance behavior, converging toward V_cap_ = 0. (blue curve in Figure 3c,d)

Although the DNO effectively induces decay‐like disturbances toward V_cap_ = 0, it increases the parameter γ in Equation (4) during implementation, leading to excessive information loss that negatively impacts the training process. The DNO keeps either N1 or N3 continuously active, thereby maintaining V_CP_ and V_CN_ (gate voltages of N5 and N6) at a high positive level, satisfying the condition (V_gsN5_ > V_thN5_, V_gsN6_ > V_thN5_). This results in additional channel capacitance formation (Figure 2a), which increases γ, accelerating weight loss. To mitigate this, we applied a standby voltage to the word line (WL) and bit line (BL) of N5 and N6, suppressing channel capacitance formation and slowing weight loss, which we named the disturbance neutralization bias (DNB). The red curves in Figure 3c,d illustrate the result under the application of DNB(1.5 V). A comparison of γ between the red and blue lines in Figure 3c,d demonstrates that the DNB effectively reduces the rate of weight loss. The results of applying a greater number of pulses according to the sequences in Figure 3a–d are shown in S3 (Supporting Information). The training results utilizing DNO and DNB are detailed in Section 2.2.

**Figure 4:**
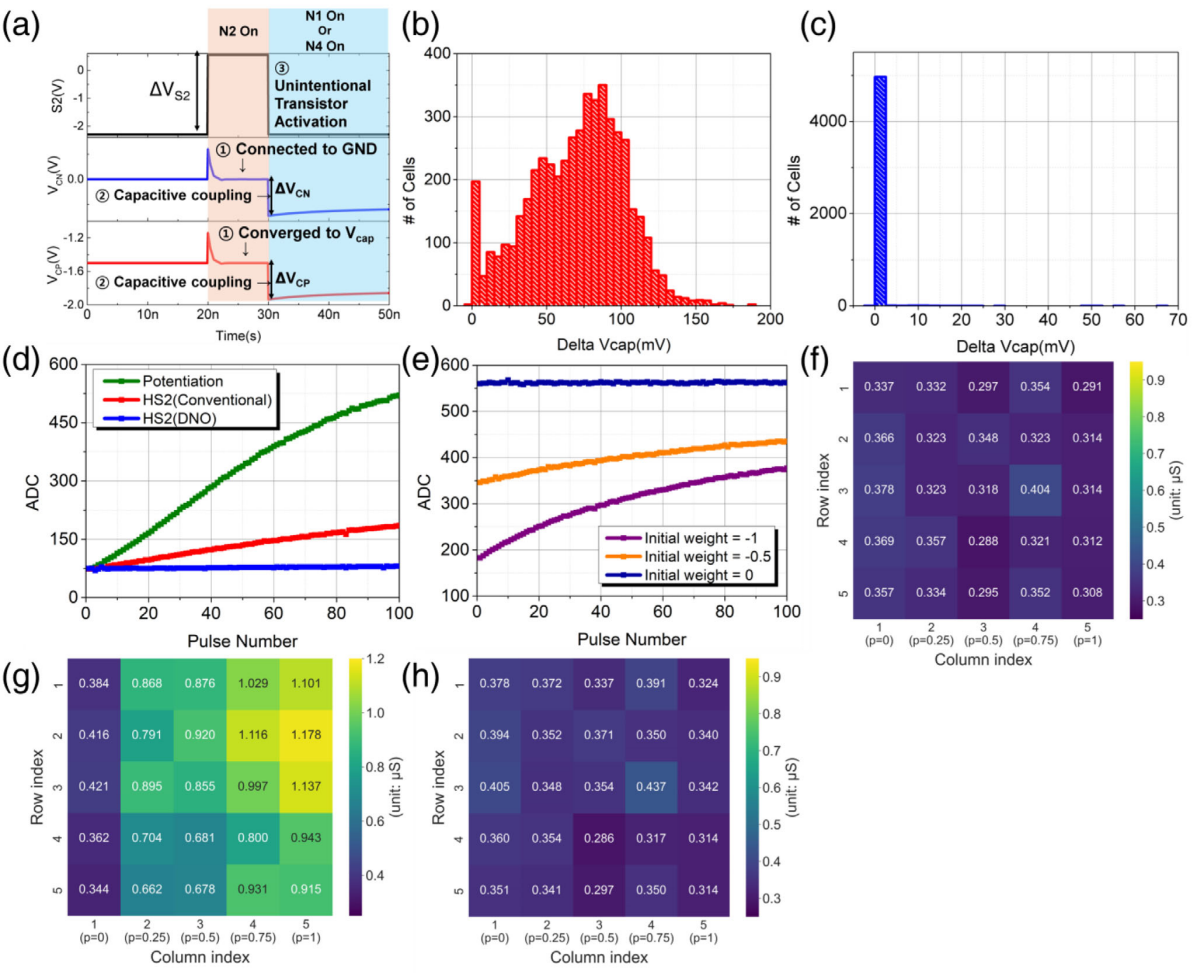
a) Mechanism of transistor selection sequence formation induced by HS2 (conventional). b) ΔV_cap_ distribution after 100 consecutive HS2 (conventional operation) under the most affected case (Initial V_cap_ = −1.5 V, DNB = 1.5 V) with a total of 5,000 cells. c) ΔV_cap_ distribution after 100 consecutive HS2 (DNO) under the same conditions as in (b). d) Weight changes in a 6T1C single cell under various transistor selection cases (Potentiation, HS2 (conventional), HS2 (DNO)). e) Weight changes after 100 consecutive HS2 (conventional) depending on the initial V_cap_. f) Initial conductance of 25 cells in a 5 × 5 crossbar array, with all cells initially in a fully depression state. g) Conductance after applying stochastic S2 pulses per column in conventional operation. The total expected number of S2 pulses is 500, meaning column 5 received 500 S2 pulses. h) Conductance after applying stochastic pulses per column in DNO. S3 pulses were applied simultaneously whenever an S2 pulse occurred.

The DNO also addresses another critical issue, unintentional transistor activation. Typically, transistor activation is determined by comparing the voltage applied between the gate and source to the threshold voltage (V_th_). However, in 6T1C, the CP and CN, which can function as the source for transistors N1‐N4, remain floating, making them uncontrollable by an external voltage. Moreover, due to the turn‐off of pulses applied to S1‐S4, the capacitive coupling effect can cause a rapid voltage drop at CP and CN, potentially triggering unintended transistor activation. When N2 or N4 is selected (HS2 or HS4 in conventional operation), it causes V_CP_ or V_CN_ to reach low voltage levels, making them particularly vulnerable to unintentional transistor activation. As shown in **Figure** **4**a, the capacitor nodes initially connect to a low GND voltage. Subsequently, during the turn‐off process of S2, the further voltage drop due to capacitive coupling reduces V_CP_ and V_CN_, potentially triggering unintentional transistor activation. If this lowered V_CP_ activates the N1 or N4 transistors, it can induce HS sequence, as previously described, leading to significant weight changes. Similarly, the reduced V_CN_ caused by the N4 selection can also trigger the transistor selection sequence. In other words, the HS2 (or HS4) under conventional operation can induce weight changes due to unintentional transistor activation. To thoroughly analyze this phenomenon, we first quantified the ΔV_CN_ and ΔV_CP_ in Figure 4a during the S2 turn‐off process shown in Equations (6) and (7) as follows:

(6) $$\Delta \;V_{CN}=\frac{C_{2}}{C_{2}+C_{3}+C_{6}}\;\times \Delta V_{S2}$$

(7) $$\Delta \;V_{CP}=\frac{C_{main}}{C_{main}+C_{1}+C_{4}+C_{5}}\;\times \frac{C_{2}}{C_{2}+C_{3}+C_{6}}\Delta V_{S2}$$

where Δ*V_CN_*, Δ*V_CP_*, Δ*V*_*S*2_ in Equation (6), (7) are illustrated in Figure 4a, while $C_{main},C_{1}∼C_{6}$ are depicted in Figure 2a. To derive Equation (6), the approximation *C_main_* = *C_main_* +  *C*_1_ +  *C*_4_ +  *C*_5_ was employed to simplify the expression, enabling a clearer illustration of the voltage drop effects caused by each parasitic component. As shown in Equations (6) and (7), these changes are highly sensitive to the device's parasitic capacitance. Furthermore, the degree of the transistor activation varies with the V_th_ value of the connected transistor. It is also important to note that by applying a DNB to control the channel capacitance of N5 and N6, the voltage drop further increases due to reduced C_5_ and C_6_, exacerbating the issue as shown in Equations (6) and (7) (S4, Supporting Information). Therefore, to analyze the impact of unintentional transistor activation, we conducted SPICE Monte Carlo (MC) simulations, considering parasitic capacitance, V_th_ variations from device measurements (S5, Supporting Information), and DNB application of the 6T1C device. Additionally, simulations were conducted considering variations in voltage and temparature, with the corresponding specifications also provided in Supplmentaty S5.

Figure 4b shows the V_cap_ changes from MC simulations after 100 consecutive HS2 (conventional operation) under DNB(1.5 V), with initial V_cap_ = −1 .5V. With V_DD_/2 = 1.5 V applied, V_CP_ reaches its initial lowest voltage when V_cap_ = −1 .5V. This initial lowest V_CP_ undergoes an additional voltage drop due to the capacitive coupling effect, representing the most affected case caused by unintentional transistor activation. As shown in Figure 4b, numerous cells undergo weight changes, with the extent of these changes varying according to the level of device variation. Detailed MC results for various initial V_cap_ and all HS cases are provided in S6 (Supporting Information). However, in the DNO, N2 (or N4) is never solely selected. As shown in Figure 2e for HS2 and HS4, (N2 and N3) and (N1 and N4) are always selected together, keeping V_CP_ and V_CN_ at high voltages near V_DD_/2. Moreover, S1 (S3) has a longer pulse width than S2 (S4), remaining on even when S2 (S4) turns off. This allows V_CP_ and V_CN_ to recover to a positive voltage, preventing unintentional transistor activation.

We validated the effectiveness of DNO against unintentional transistor activation through SPICE MC simulations and device measurements. Figure 4c shows that no issues arise even in the most affected cases (Initial V_cap_ = −1.5 V, DNB = 1.5 V) during repeated HS2 (DNO) cases in the MC simulation. Figure 4d validates this through device measurement, demonstrating that the issue was fully controlled via DNO in the same case (Initial V_cap_ = −1.5 V, DNB voltage = 1.5 V).

Additionally, Figure 4e shows varying weight change tendencies due to repeated HS2 (conventional operation), depending on the initial V_cap._ This confirms that as V_cap_ decreases, the lower initial V_CP_ increases the device's susceptibility to unintentional activation in measurements. We also validated our approach on a 5 × 5 crossbar array under a stochastic scheme, assigning different S2 pulse probabilities to each column. Figure 4f shows the initial conductance of the 25 cells. Figure 4g,h compares conductance changes when pulses were applied under conventional operation and DNO. In the conventional operation (Figure 4g), conductance increased with higher pulse probabilities, while the DNO (Figure 4h) maintained the same conductance across all columns, effectively mitigating the write disturbance caused by unintentional transistor activation.

### 2.2 Disturbance‐Aware On‐Chip Training Simulation

To evaluate the effectiveness of the aforementioned approaches in actual learning, we conducted a simulation that incorporates the effects of all possible transistor selection cases in the device array. Specifically, we quantified the potential changes at the V_CP_ and V_CN_ for all selection cases using circuit simulation with a single scenario. Notably, in the 6T1C, while the voltages at other nodes are defined by the external voltage, only the CP and CN nodes remain undefined. In other words, by creating a lookup table recording these node changes for all possible cases, we can fully capture the device's real‐time response during both training and inference, including non‐ideal disturbance behaviors, ultimately enabling precise disturbance‐aware training simulations. (Details in S7, Supporting Information). Using this lookup table, we developed a simulation to evaluate a 6T1C‐based AI accelerator and assess the impact of the disturbances. Training was conducted using the rTT, as presented in our previous work.^[^ ^25^ ^]^ (Details in the Experimental section.)

Evaluation of the training capability of the 6T1C array revealed that, owing to the application of DNO and DNB, learning proceeded smoothly in the fully connected layer (FCL) (S8, Supporting Information). However, despite using the proposed methods, challenges persist in the convolutional layer (CL) (S8, Supporting Information). This discrepancy arises from differences in the update mechanism. In the CL, a single‐weight kernel processes one data sample multiple times as it strides across. As a result, CL training demands a minimum number of update pulses equal to the number of strides per image (weight sharing), unlike FCL, where only one update pulse is required per data sample. This leads to a significant increase in the number of HS cases, referring to the cells in HS1 through HS4 in Figure 2e. Consequently, the high frequency of decay‐like disturbance occurrences in the CL results in substantial weight loss, preventing the achievement of optimal learning accuracy.

**Figure 5:**
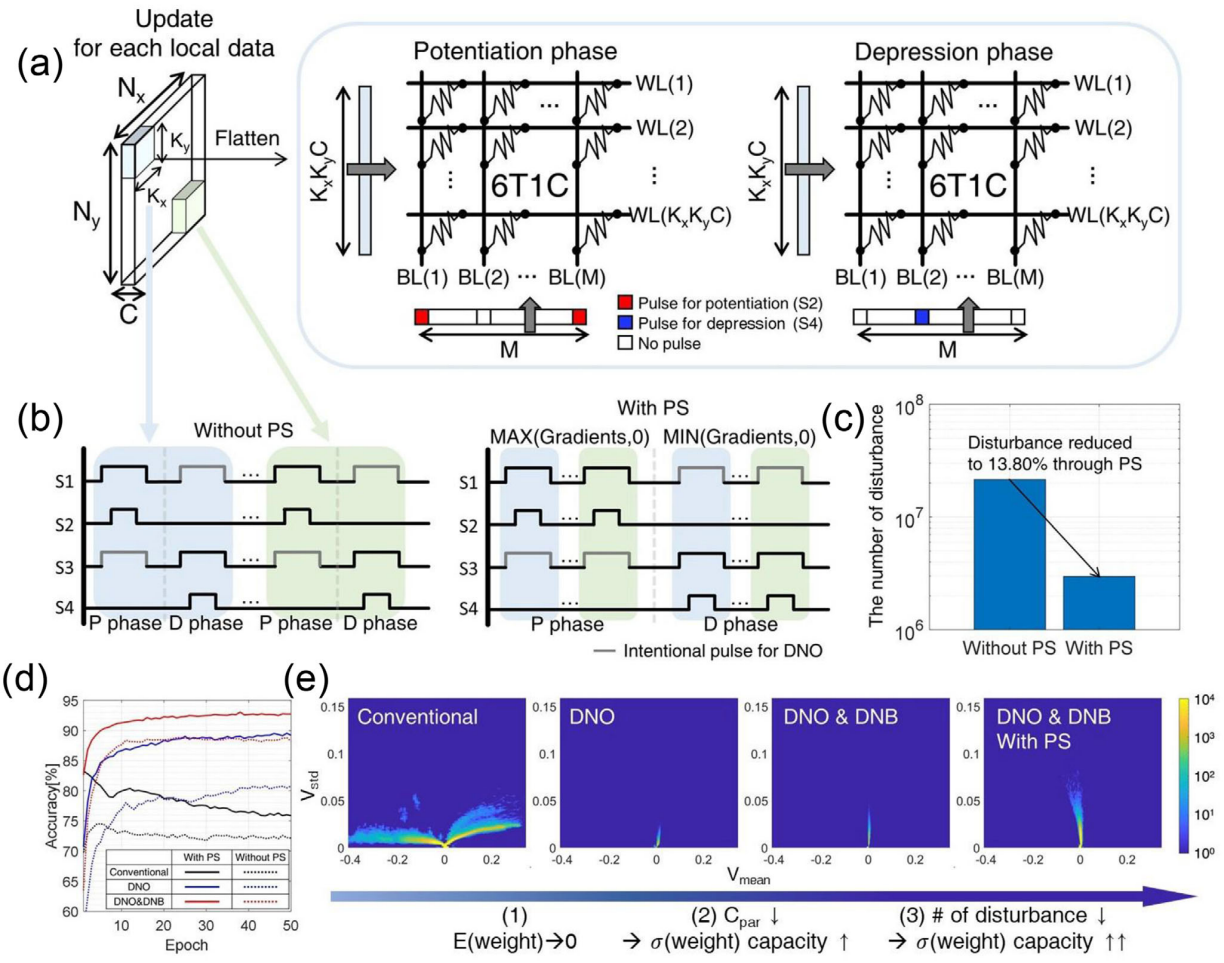
a) A schematic of pulse scheduling during the training of a convolutional layer. In the traditional approach, the potentiation phase(P phase) and depression phase(D phase) are repeatedly performed for each region processed by the convolutional kernel to update the data. b) Through pulse scheduling, potentiation and depression phases are divided and performed across the entire area. c) Reduction in the number of decay‐like disturbance events under DNO and DNB conditions with PS applied. d) CIFAR10 training performance before and after the application of DNO and DNB. The solid and dashed lines represent the cases with and without pulse scheduling (PS), respectively e) Distribution of V_cap_ during the entire training process before and after the disturbance neutralization operation, as well as the application of disturbance neutralization bias voltage and PS. The mean and standard deviation of V_cap_ in each 6T1C form the grid points.

To address this issue, we developed the additional pulse scheduling (PS) technique for training the 6T1C array mapped to CL. In a typical deep neural network, the positive values resulting from batch normalization followed by ReLU activation are passed as inputs to the next layer.^[^ ^30^, ^31^, ^32^ ^]^ Therefore, the sign of the delta weight is determined by the sign of the gradient during updates (**Figure** **5**a). Since the gradient generally contains both positive and negative elements, the potentiation and depression phases are performed separately to enable fully parallel updates. Specifically, in the 6T1C, where the transistors responsible for potentiation (N1, N2) and depression (N3, N4) are distinct, the conventional approach (Without PS in Figure 5b) that sequentially processes data samples through alternating potentiation and depression phases results in repeated selection of the transistors connected to the CP and CN nodes. This significantly increases the occurrence of decay‐like disturbances toward V_cap_ = 0 during CL training.

Therefore, instead of sequentially updating the information of the local regions, we separated the positive and negative gradients from all the information contained within a single data sample (With PS in Figure 5b). Then, we applied all the update pulses corresponding to the positive gradients, followed by the update pulses for the negative gradients. This simple scheduling technique consumes the same update time while significantly reducing the impact of decay‐like disturbances, thereby improving the learning performance of 6T1C arrays mapped to the CL. To precisely analyze the effect of DNO, DNB, and PS, we trained the second layer of ResNet18, which is one of the layers with the highest degree of weight sharing, from scratch while keeping all other parameters fixed using pretrained values. As shown in Figure 5c, the application of the PS method significantly reduces the occurrence of decay‐like disturbances during training. Figure 5d presents the training performance with and without the application of DNO, DNB (1.5 V), and PS, demonstrating their impact on training performance. As indicated by the red solid line in Figure 5d, even with the implementation of disturbance‐aware training that accounts for all disturbances in real time, applying all three proposed methods achieves optimal learning accuracy of ≈93%, even in convolutional layer where a single image requires 1024 pulses for potentiation and 1024 pulses for depression, leading to frequent disturbances by HS cases during training. To further clarify the effectiveness of the proposed schemes, we extracted the range of V_cap_ used during training (Figure 5e). As shown in Figure 5e, the DNO minimized unintended V_cap_ divergence. Furthermore, by minimizing γ through DNB and reducing the occurrence of decay‐like disturbances to 13.80% via pulse scheduling, the range of V_cap_ used during training was extended 3x. This minimized idle memory state during training, resulting in improved learning accuracy. The three proposed methods effectively mitigate encountered issues and achieve remarkably high learning accuracy without increasing the complexity of the peripheral circuits or extending training time, offering a highly efficient and practical solution for implementation.

### 2.3 Analysis of Cell Size Reduction and Energy Consumption

In the previous Section 2.2, we demonstrated that the three methods we devised successfully addressed the disturbance caused by HS cases in both FCL and CL, achieving high learning accuracy. In Section 2.3, we analyzed these methods regarding cell area and energy consumption.

#### 2.3.1 Cell Area

Another challenge associated with synaptic devices based on capacitors is the issue of large cell areas resulting from the large capacitor sizes. In particular, as described in Section 2.1, for 6T1C, the ratio between the main capacitance and the parasitic capacitance, which is represented by the parameter γ, determines the performance of analog in‐memory computing. This presents a difficulty in reducing the size of the main capacitor, ultimately hindering improvements in integration density.

**Figure 6:**
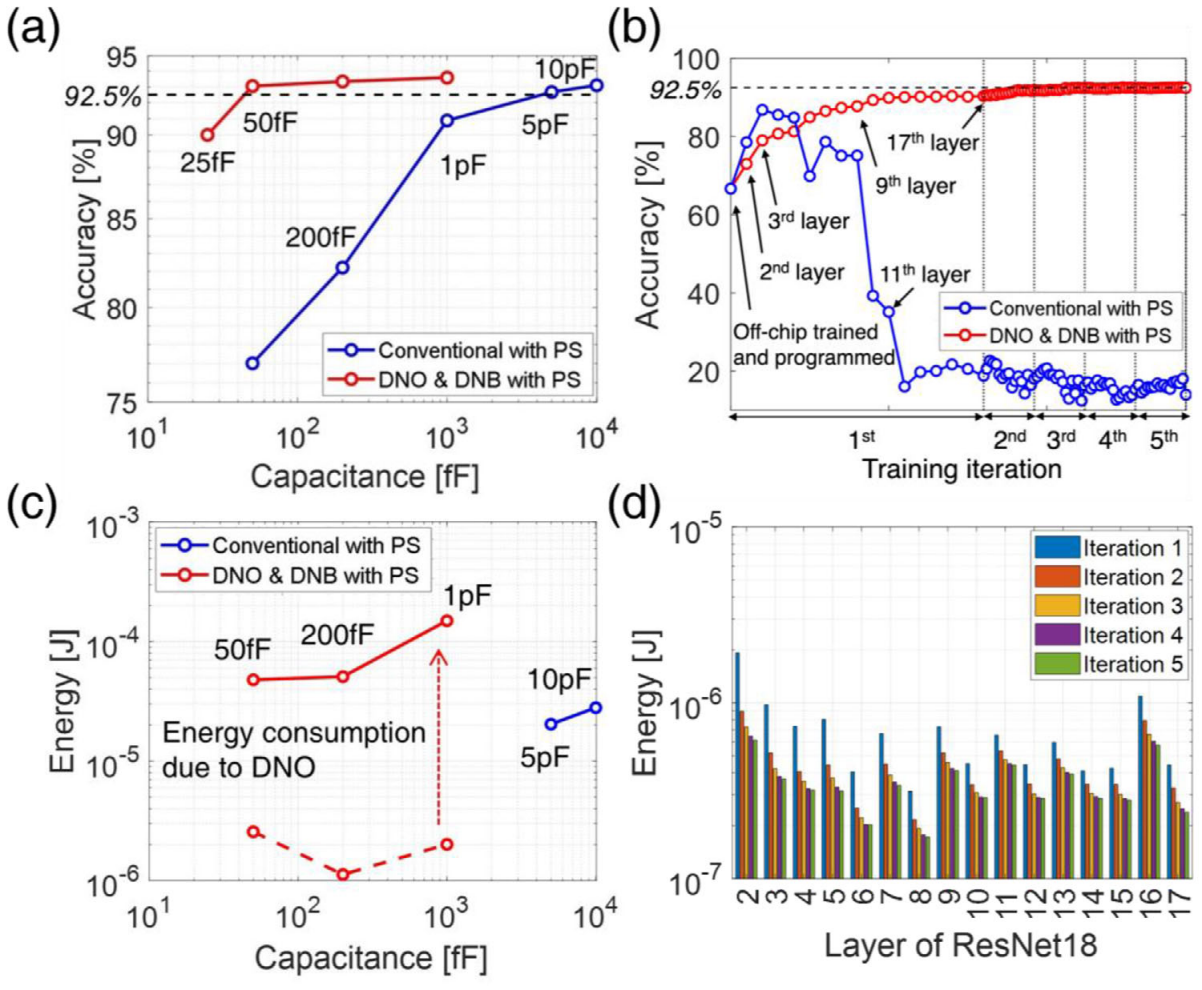
a) CIFAR‐10 training performance depending on the capacitance of the 6T1C device and the use of DNO/DNB schemes, when only the second layer of the ResNet18 neural network is trained from scratch. b) Sequential fine‐tuning of the 2^nd^ to 17^th^ layers of ResNet18 via on‐chip training with 50fF 6T1C. c) Energy consumption of the 6T1C array (Number of synaptic cells = 36,864, number of strides per image = 1,024) for performing outer product operations until reaching the threshold accuracy in (a). d) Energy consumed by the 6T1C arrays mapped to each layer during the on‐chip training process in (b) for performing outer product operations.

We investigated the effectiveness of our proposed methods by performing simulations with various main capacitor sizes, using the parasitic capacitance levels of IGZO TFTs from published research.^[^ ^33^, ^34^ ^]^ For smooth CL training, the PS method was applied throughout, and only the effect of DNO and DNB (1.5 V) was analyzed. One of the layers with the highest frequency of weight sharing, the second layer, was trained from scratch, and the results are presented in **Figure** **6**a. In conventional operation, optimal learning accuracy (>92.5%) was achieved at a capacitance level above 5 pF, and the accuracy sharply declined as the capacitance decreased. However, when applying DNO and DNB, the learning performance exhibited relatively robust behavior against capacitance reduction, achieving optimal accuracy even when the capacitance was reduced to 50fF (Figure 6a), comparable to the capacitance level of a DRAM cell.^[^ ^29^ ^]^ Building upon the confirmation of training efficacy of 50fF, we trained ResNet18 from the 2^nd^ layer to the 17^th^ layer using the rTT with 50fF 6T1C, as shown in Figure 6b. The first and last layers were excluded from training, following prior studies,^[^ ^33^, ^34^ ^]^ and pretrained parameters were mapped onto the NVM array implemented via AIKHKIT. Mini‐batch statistics and batch normalization parameters were kept fixed to evaluate the extent to which the entire network could be trained solely through on‐chip training. Training was conducted sequentially from the 2^nd^ layer onward,^[^ ^35^ ^]^ with both inference and backpropagation performed directly on the NVM arrays where weights were mapped. Although network performance initially degraded by ≈67% immediately after mapping, five iterations of fine‐tuning enabled performance recovery to ≈93%.

#### 2.3.2 Power Consumption

Although the DNO described in Section 2.1 effectively addresses the disturbance issue in HS cases, it intentionally flows current through transistors N2 and N3 or N1 and N4, resulting in additional energy consumption. The effectiveness of the DNO was validated by analyzing the energy consumption relative to training performance. The energy consumption for all possible transistor selection cases during training was compiled into an energy look‐up table based on the V_CP_ and V_CN_ states mentioned in Section 2.2, and the total energy used to achieve the optimal accuracy of 92.5% was analyzed (Details in S10, Supporting Information). Notably, the energy analysis was conducted exclusively for the update operation, excluding the read operation. As shown in Figure 6c, it was confirmed that the energy consumption of 50 fF, optimized through the DNO, is similar to the energy consumption of 5 to 10 pF, which is the minimum capacitance level required to maintain optimal accuracy in conventional operation. This is attributed to the fact that, although unwanted currents increase energy consumption in the DNO, reducing the capacitance size enables a reduction in the overall current level required for programming. Moreover, as shown in Figure 6d, all layers from the 2^nd^ to the 17^th^ exhibited significantly low energy consumption levels (on the order of 10^−7^–10^−6^ J) for outer product operations during the training process. This phenomenon is attributed to the decreasing number of weight sharing as the layer size increases. Furthermore, it was observed that as training progresses, the gradient magnitude decreases, leading to a reduction in energy consumption. Consequently, the proposed methods have paved the way for reducing cell size while maintaining the same level of energy consumption and learning performance.

## 3 Conclusion

We have reported a thorough analysis of the write disturbance mechanism in IGZO TFT and capacitor‐based 6T1C synaptic devices, alongside the development of simple and improved operational schemes (DNO, DNB) that effectively address this issue. The 6T1C device offers reliable endurance and device variation characteristics. However, due to the parasitic capacitances, it suffers from write disturbances in half‐selected cases, which become more pronounced as the capacitor size decreases. However, the proposed schemes serve to neutralize both the directionality and magnitude of write disturbances in half‐selected 6T1C cells, and we have validated their effectiveness in synaptic array measurements. Furthermore, a pulse scheduling method was devised based on the characteristics of 6T1C, which are affected differently by disturbances depending on the sequence of update pulses. Ultimately, by combining the proposed three disturbance‐aware on‐chip training schemes, we conducted a simulation that accurately reflects real‐time disturbances in CNN architectures requiring a high number of update pulses, thereby achieving software‐equivalent accuracy on the CIFAR‐10 dataset. Additionally, the proposed schemes reduced the required capacitor size for optimal learning accuracy by more than ∼100 times compared to conventional on‐chip training methods, achieving a cell capacitance of 50fF, which is comparable to DRAM, effectively addressing the cell density limitation of capacitor‐based synaptic devices. This approach, combined with the inherent advantages in endurance and device variation, opens a realistic path for hardware‐based deep learning.

## 4 Experimental Section

### Device Fabrication

The device fabrication process is identical to that described in previously published studies.^[^ ^25^ ^]^ The synaptic array, consisting of IGZO TFTs and capacitors, was built on a silicon substrate with a multi‐metal layer stack. A 200Å tungsten layer was deposited as the lower capacitor electrode, followed by photolithography and dry etching to pattern the electrode and its wiring. Then, a high‐k oxide layer, serving as the capacitor insulator, was deposited using atomic layer deposition. A VIA for connecting the lower and upper electrodes was formed through wet etching and photolithography. Subsequently, a 200Å tungsten layer was deposited as the upper capacitor electrode, patterned to align with the lower electrode.

An oxide underlayer for the IGZO TFT was then deposited, and VIA formation was performed to connect the upper capacitor electrode to the IGZO TFT source/drain. The source/drain was created by depositing and etching a 200 Å tungsten layer. IGZO, the channel material, was sputtered to a 100 Å thickness in an RF plasma, and patterned by photolithography and wet etching. Finally, the upper gate electrode of the IGZO TFT was deposited with tungsten, completing the synaptic array structure through dry etching and photolithography.

### Experimental Setup for Device Measurement

The experimental setup for device measurement is identical to that described in previously published studies.^[^ ^25^ ^]^ The synaptic measurement system utilized a PCB that incorporated a MCU alongside discrete components for the peripheral circuit. To connect with the synaptic cells on an 8‐inch wafer, a 45‐pin probe card connected to an Eg4090 was employed, while the required voltages were delivered to the PCB through a DC power supply. Input signals, such as pulse width and repetition count, were sent from a personal computer, which communicated with the MCU via a universal asynchronous receiver transmitter.

### SPICE Simulation

The circuit simulation of the 6T1C device was conducted using HSPICE, with the fitting of a single IGZO TFT performed prior to synapse‐level simulations. Since HSPICE does not have a model for IGZO TFTs, the key electrical characteristics of the IGZO TFTs were emulated by modifying the main parameters of the RPI a‐Si TFT model (level 61) from the HSPICE MOSFET models. The parasitic capacitance, which is critically important in this study, was set based on a published paper that measured the parasitic capacitance components of IGZO TFTs.^[^ ^36^, ^37^ ^]^ After implementing the single IGZO TFT, the 6T1C synapse‐level simulations were conducted with a realistic and manufacturable device size of channel length = 0.4μ m, channel width = 0.4 µm. To account for the effects of half‐selected cases in the 6T1C device, a comprehensive look‐up table was created to track the changes in the two undefined nodes, V_CP_ and V_CN_, for all possible transistor selection cases Potentiation (N1, N2 selection) Depression (N3, N4 selection), N1 selection, N2 selection, N3 selection, N4 selection, (N2, N3) selection in DNO, (N1, N4) selection in DNO, Pre‐read N5, Post‐read N5, Pre‐read N6, Post‐read N6). This look‐up table enabled to determine the device's response to all selection cases that could occur during training. Using the SPICE‐generated look‐up table, software simulations were subsequently carried out.

### 6T1C Array Simulation for DNN Training

To accurately capture the physical behavior of 6T1C devices under pulse‐based updates during on‐chip training, the open‐source AIHWKIT^[^ ^38^ ^]^ framework was extended with a custom simulation backend. The implementation was primarily integrated into the kernel‐level CUDA update path, including *pwu_kernel.h*, *pwu_kernel_parameter.h*, *rpucuda_6T1C_device.h*, and *rpucuda_6T1C_device.cu*. In the default AIHWKIT implementation, the number of pulse coincidences between forward and backward pulse directions, along with the potentiation or depression phase, is computed using the *getNfromCount* function defined in *pwu_kernel.h*. The update operation is then applied fully in parallel across all devices in the crossbar array, leveraging CUDA kernel functions such as *__global__ void update functor*. The magnitude of each update is determined by a device‐specific update functor struct, which is defined in the corresponding.cu file for each device type. In the work, however, the effect of temporal ordering of pulse sequences was emphasized due to the nontrivial disturbance behavior intrinsic to 6T1C devices. Therefore, a new bit‐wise pulse decoding function (*getHSCount*) was introduced to extract not only the sequence but also the directionality and polarity of each pulse over time. Instead of applying bitwise operations (such as the AND operator used in the original *getNfromCount* function) to compute pulse coincidences, the raw pulse bit streams were directly utilized and retrieved via *getXData* and *getDData* functions defined in *bit_line_maker.h* of the original AIHWKIT. These streams, originally formatted as uint32_t or uint64_t values, were parsed to isolate binary representations indicating the occurrence of pulses, with polarity information intentionally omitted. The excluded polarity bits were subsequently utilized to infer the corresponding synaptic update phase, either potentiation or depression. The determination of potentiation and depression phases was implemented identically to AIHWKIT, by deriving the *negative* variable in the same manner as in the original framework, with no changes introduced to this mechanism. This enables a precise reconstruction of pulse sequences at each crosspoint during training. To integrate this temporal resolution into CUDA kernels, a new update kernel was implemented *(__global__ void update functor for 6T1C*), which distinguishes pulse conditions based on both phase (potentiation and depression) and direction (forward and backward). Specifically, the following six cases were encoded. During the potentiation phase: A forward‐only pulse triggers activation of the N1 path (Case 1). A backward‐only pulse activates the N2 path (in the case of DNO, N2 and N3 paths) (Case 2). Coincidence of forward and backward pulses leads to co‐activation of both N1 and N2 paths (Case 3). During the depression phase: A forward‐only pulse activates the N3 path (Case 4). A backward‐only pulse activates the N4 path (in the case of DNO, N1 and N4 paths) (Case 5). Coincidence of forward and backward pulses leads to co‐activation of both N3 and N4 paths (Case 6). These cases were passed to the *__device__ __forceinline__ void operator* in *update functor struct* defined in *rpucuda_6T1C_device.cu*, which inherits from AIHWKIT's *PulsedRPUDeviceCuda* class, for updating device states. In this class, three *CudaArray<T>* instances were defined and instantiated by *std::unique_ptr* objects with array size dimensions: one to store the voltages at the top floating nodes(CP node) of the 6T1C devices in the array, one for the bottom floating node(CN node) voltages, and one for the voltage difference between the two nodes, corresponding to the V_cap_. The update logic leverages precomputed 2D lookup tables for each of the defined pulse cases. These include voltage shifts at the floating nodes in response to potentiation, depression, N1 HS, N2 HS, N3 HS, N4 HS, N2 and N3 DNO, N1 and N4 DNO, pre‐read N5 (activating N3 for CN node boosting), post‐read N5 (deactivating N3), pre‐read N6 (activating N1 for CP boosting), and post‐read N6 (deactivating N1). In total, 24 distinct voltage shift tables were defined within the 6T1C device class (Details in S7, Supporting Information). To evaluate energy consumption associated with outer product operations, 8 energy lookup tables were also included (Details in S10, Supporting Information). These include potentiation, depression, N1–N4, and the DNO cases (N2 and N3, N1 and N4). The impact of DNB was investigated by selecting condition‐specific tables based on DNB conditions. Each table spans a 2D domain from −2.4 to 3.0 V in both axes (CP and CN node voltages), discretized with 0.1 V resolution, totaling 3025 data points per table. These boundaries were selected based on circuit simulations confirming that the floating node voltages never exceed this range under any realistic condition. The initial values of the CP and CN nodes are set to V_DD_/2 (1.5 V), and updated voltages of floating nodes are used to index into the corresponding table using bilinear interpolation. Tables are stored on the GPU using *CudaArray<T>* initialized in the constructor of the *RPUDeviceCuda* class of 6T1C. During the read operation for transfer purposes in rTT, the weight is computed by normalizing the conductance of the read transistor with respect to the voltage of the floating nodes under either the N3 activation (CN node boosting) or N1 activation (CP node boosting) condition. Following the read access, the voltage shifts due to deactivating N3 or N1 are also incorporated to update the internal device state. The *run* function in the *PWUKernelParameter* class, located in *pwu_kernel_parameter.h*, invokes the full update process. It launches CUDA kernels with all required parameters, including 6T1C‐specific voltage and energy lookup tables, passed via pointer arguments. Through this *run* function, a device‐specific update is executed by invoking the *__global__ void update functor* defined in *pwu_kernel.h*, which in turn calls the *__device__ __forceinline__ void operator* implemented in the corresponding *update functor struct* defined in *rpucuda_6T1C_device.cu*. This invocation chain mirrors the architectural structure employed in the original AIHWKIT framework, thereby preserving compatibility and consistency in kernel execution flow across device types. In particular, this design maintains seamless integration with core components of the original update pipeline, such as void *RPUCudaPulsed<T>::updateMatrixIterator* in *rpucuda_pulsed.cu* and *void PulsedWeightUpdater<T>::update* in *pulsed_weight_updater.cu*, ensuring that the extended 6T1C update process adheres to the modular and extensible architecture of AIHWKIT without compromising interoperability. As for pulse scheduling, the logic was implemented in the *__global__ void update functor for 6T1C* within the *pwu_kernel.h*. Based on the potentiation or depression phase as determined by *getHSCount*, pulses were scheduled in two stages: first, all reactions corresponding to the potentiation phase were applied, followed by those related to the depression phase. The sequential scheduling mechanism is described in the pseudocode provided in S9 (Supporting Information).

The performance of the 6T1C arrays was evaluated by training on the MNIST dataset using a 2‐layer MLP and a 3‐layer CNN, and on the CIFAR‐10 dataset using a ResNet18 neural network. When mapping devices to a neural network for training, the rTT algorithm was employed. For instance, the core array was mapped to an NVM array implemented using AIHWKIT, while the auxiliary array for updates was mapped to the 6T1C array. For the NVM devices in AIKHKIT, the parameters were set as follows: *E*(|Δ*w*|) = 0.002, NL (Non‐Linearity) = 1.0 in Equation (8) and 30% cycle‐to‐cycle standard deviation, and 6% device‐to‐device standard deviation for *E*(|Δ*w*|), NL, and conductance range. In Equations (8) and (9), w is weight, G is conductance, and G_ref_ is the conductance corresponding to weight 0. Additionally, a 6% current sum standard deviation was applied in both the inference and weight transfer processes. To enhance the effective weight resolution of analog devices during mapping, a scaling factor (SF) similar to the prior study was applied.^[^ ^39^ ^]^ Typically, weight distributions in neural networks follow a Gaussian distribution with a mean of zero and a standard deviation significantly smaller than one. By fitting the useful weight range to the conductance range of the analog device, effective weight resolution was improved (Equation (10)). In Equation (10), x is input, o is output, V is pulse height, PW is pulse width. As shown in Equation (11), an SF is assigned for each ResNet18 layer, optimizing the device mapping process while accounting for device‐to‐device conductance range variations in the NVM by normalizing *MAX*(|*w_pretrained_* |) values by 0.8

(8) $$\begin{array}{c} \Delta w=\left(1-sign\left(\Delta w\right)\times NL\;\times \;\frac{G-G_{\mathrm{ref}}}{\left(G_{\max}-G_{\min}\right)}\right)\times sign\left(\Delta w\right)\times E\left(\left|\Delta w\right|\right) \end{array}$$

(9) $$w=\frac{G-G_{\mathrm{ref}}}{0.5\left(G_{\max}-G_{\min}\right)}\;$$

(10) $$\begin{array}{ccc} o_{j} & = & \sum_{i}x_{i}w_{ij}=SF\;\sum_{i}x_{i}\frac{w_{ij}}{SF}=SF\sum_{i}x_{i}\;w_{ij,mapped}\\ & & =SF\sum_{i}V*PW_{i}\left(\frac{G_{ij}-G_{ref}}{0.5(G_{max}-G_{min})}\right) \end{array}$$

(11) $$SF=\frac{MAX\left(\left|w_{pretrained}\right|\right)}{0.8}$$

### Retention‐Centric Tiki Taka Algorithm (rTT)

The Tiki‐Taka algorithm consists of two systems: the core array, which performs inference, and the auxiliary array, which is responsible for updates at each layer of a neural network. Inference and backpropagation are executed within the core array, after which the auxiliary array is updated based on the input and gradient information. Periodically, a column or row from the auxiliary array is read and used to update the corresponding column or row in the core array, thereby transferring the learned information. To mitigate conductance convergence caused by update asymmetry in the analog devices of the auxiliary array, the conductance convergence point termed the symmetry point (SP) is used as a reference. During the transfer process, this reference value is subtracted to compensate for conductance convergence, effectively serving as both a correction mechanism and a form of regularization. Leveraging these characteristics of the Tiki‐Taka algorithm, th**e** rTT method was developed to further address retention and disturbance‐induced conductance convergence. The 6T1C device enables linear and symmetric readout of stored information by performing differential sensing. Specifically, by turning on transistor N3 and reading N5, then turning on transistor N1 and reading N6, the information stored in the capacitor can be extracted in a linear and symmetric manner by subtracting the latter from the former. However, if transistor N1 is turned on while reading N5, and transistor N3 is turned on while reading N6, subtracting these values would allow access to the zero‐state information without loss of stored charge. This capability eliminates the need for the explicit SP identification and reference storage required in the conventional Tiki‐Taka algorithm. Instead, each device within the 6T1C array can autonomously determine a stable reference without additional reference arrays. Moreover, the conductance at V_cap_ = 0 inherently represents the equilibrium state under leakage‐induced and disturbance‐induced convergence. Consequently, the rTT algorithm not only compensates for conductance convergence following the original Tiki‐Taka principle but also utilizes this effect as an implicit form of regularization. All simulations in this study employed the rTT algorithm, where the core array was implemented using AIHWKIT‐based NVM, while the auxiliary array was modeled based on the characteristics of the 6T1C device.

## Conflict of Interest

The authors declare no conflict of interest

## Supporting information



Supporting Information

## Data Availability

The data that support the findings of this study are available from the corresponding author upon reasonable request.
