# Supplementary material for: Disturbance‐Aware On‐Chip Training with Mitigation Schemes for Massively Parallel Computing in Analog Deep Learning Accelerator
Source: Adv Sci (Weinh). 2025 May 20;12(23):2417635. doi: 10.1002/advs.202417635 (PMC12199431; doi:10.1002/advs.202417635)
Supplement: Supplementary file 1 — Supporting Information [file ADVS-12-2417635-s001.pdf]

ADVANCED SCIENCE
Open Access

Supporting Information

for *Adv. Sci.*, DOI 10.1002/advs.202417635

Disturbance-Aware On-Chip Training with Mitigation Schemes for Massively Parallel Computing in Analog Deep Learning Accelerator

Jaehyeon Kang, Jongun Won, Narae Han, Sangjun Hong, Jee-Eun Yang, Sangwook Kim and Sangbum Kim**

Supporting Information

Disturbance-aware On-chip Training with Mitigation Schemes for Massively Parallel Computing in Analog Deep Learning Accelerator

Jaehyeon Kang, Jongun Won, Narae Han, Sangjun Hong, Jee-Eun Yang, Sangwook Kim, and Sangbum Kim**

S1. Comparison of this study with other device-algorithm-based studies

Recently, numerous studies have demonstrated the feasibility of hardware-based training by compensating for the inherent non-idealities of hardware through appropriately designed training algorithms.[1-4] Notably, recent studies on the integration of RRAM with advanced algorithms, such as c-TTv2 and AGAD,[4] have effectively addressed various device-level challenges – including weight update asymmetry, limited multi-level states, reference conductance offset issues, and the need for additional arrays to store reference conductance – thereby demonstrating the feasibility of hardware-based training. Supplementary Information S1 aims to compare previous device-algorithm optimization studies with the present work.

While effective training algorithms can alleviate some of the stringent hardware requirements, certain limitations remain difficult to overcome through algorithmic approaches alone. For instance, as shown in Figure S1, CMOS compatibility and endurance cannot be easily compensated for by algorithms.

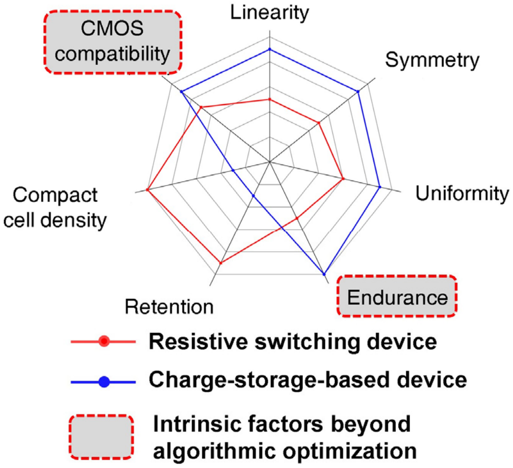

Figure S1. Spider chart illustrating the key characteristics required for synaptic devices in neural network training. As indicated in the figure, CMOS compatibility and endurance remain challenging to overcome, even with algorithmic assistance.

From this perspective, the 6T1C device, which is composed of well-established materials such as capacitors and oxide semiconductors, offers significant advantages. Extensive research on monolithic 3D (M3D) integration of CMOS and IGZO TFTs has already demonstrated excellent CMOS compatibility.[5,6]

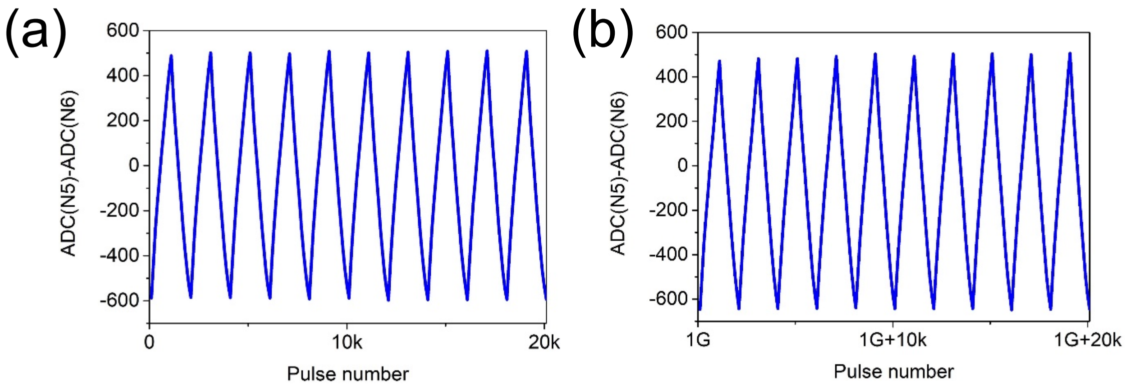

Figure S2. (a) Measurement results in the initial cycle in 6T1C (b) Measurement result after applying 10^9 update pulses (update pulse height was 0.5V/-2V and length was 1μs)

More importantly, the superior endurance characteristics of charge-storage synaptic devices, including 6T1C, compared to resistive switching devices, make them highly suitable as synaptic elements for on-chip training. Figure S2 from our previous study[7] demonstrates that even after the application of 10^9 pulses, the ADC value remains nearly unchanged, highlighting the outstanding endurance of the 6T1C device. On the other hand, RRAM, which relies on an atomic-level conductance modulation mechanism, inherently suffers from poor endurance characteristics. Despite distinguishing only between the high-resistance state (HRS) and low-resistance state (LRS) at the array level, RRAM has been reported to exhibit an endurance of approximately $10^5 \sim 10^7$ pulses.[8,9]

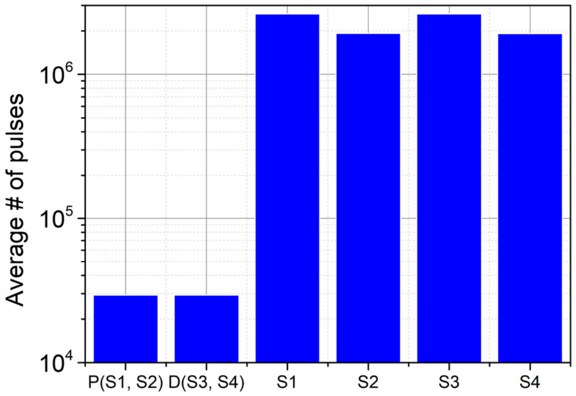

Figure S3. The average number of pulses applied per 6T1C cell in the second convolutional layer during Resnet18 training, until the optimal training accuracy is achieved.

Figure S3 shows that the average number of pulses applied per 6T1C cell in the second convolutional layer during Resnet18 training, until the optimal training accuracy is achieved. S1-S4 correspond to the N1-N4 gates, respectively. The results indicate that achieving the optimal training accuracy requires an exceptionally large number of pulses, exceeding 10^6. Furthermore, more complex neural networks and training datasets are expected to demand an even greater number of pulses. Given these situations, the 6T1C cell, with its significantly superior endurance compared to NVM, is well suited for training.

Furthermore, beyond the intrinsic advantages of the 6T1C device itself, its benefits can be further amplified when combined with the retention-centric Tiki-Taka algorithm (rTT)[7] and the DNO, DNB and PS methods proposed in this study for array operation. For auxiliary devices in Tiki-Taka algorithm, it is crucial to enable fast write operations using an open-loop approach rather than a closed-loop method based on precise program-and-verify processes. As a result, disturbances frequently occur within the array during the update process, making error correction particularly challenging. This study is the first to address the issue of write disturbance- an aspect not considered in prior research- by leveraging the regularization effect of the Tiki-Taka algorithm.

The 6T1C device enables highly linear weight updates, and due to its structural characteristics, the expected conductance convergence point (G_{sym}) in the presence of update asymmetry corresponds to the conductance at $V_{cap} = 0$.[7] Notably, this is identical to the disturbance convergence point($G_{disturb}$) of pulses applied through the proposed DNO method. As shown in Figure S4, measurement results from 30 cells indicate that the difference between the two convergence points (G_{sym} and $G_{disturb}$), when converted into weight values, follows a Gaussian distribution with a mean of -0.0076 and a standard deviation of 0.0221.

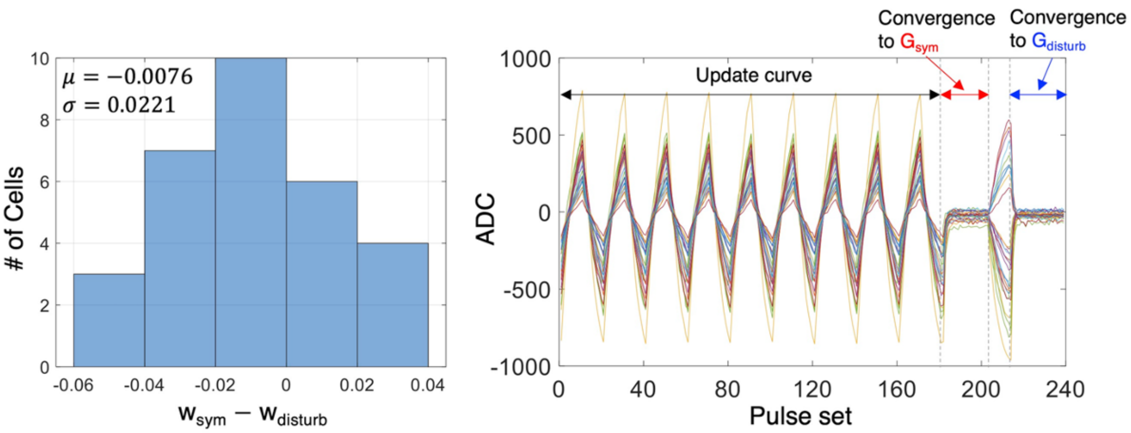

Figure S4. Distribution of the difference between G_{sym} and $G_{disturb}$ extracted from 30 cells of the 6T1C array

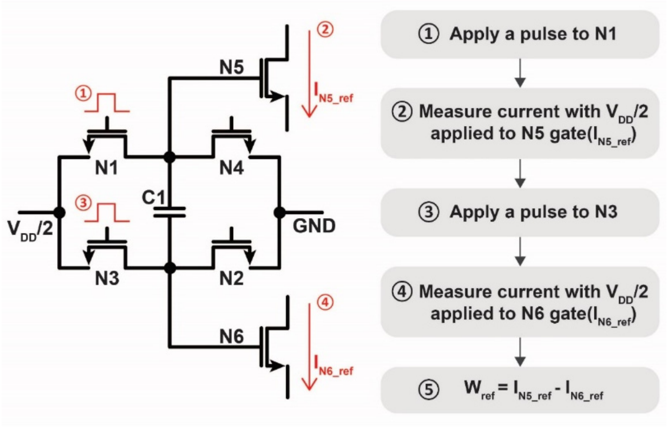

Figure S5. Method for reading the reference conductance in the 6T1C device without reference array

This characteristic minimizes the convergence effect caused by asymmetry while allowing the conductance at $V_{cap} = 0$ to be set as a reference conductance. Consequently, this approach can mitigate the convergence effects induced by disturbance and retention while utilizing them for regularization. Furthermore, as shown in Figure S5, the conductance at $V_{cap} = 0$ can be easily read without information loss from capacitors. This method provides individual referencing for all cells within the array without requiring an additional reference array, offering a significant advantage.

Additionally, integrating the c-TTv2[4] with the approach proposed in this study is expected to further enhance stability. Previous research has demonstrated that the performance of c-TTv2 can be influenced by the number of states in the analog device. In the case of 6T1C, this can be readily optimized by adjusting the pulse width. Moreover, the core function of c-TTv2 – sign inversion via the chopper – can be conveniently implemented in the 6T1C structure by applying signals to S3 & S4 for potentiation and to S1 & S2 for depression (compared to the conventional method using S1 & S2 / S3 & S4). If it is necessary to reset the conductance of the auxiliary device before sign inversion, the 6T1C device allows for straightforward initialization to the reference value by simultaneously activating S1 & S3 or S2 & S4. By combining the digital low-pass filter and chopper-based inversion method of c-TTv2 with the DNO, DNB, PS, and rTT methods proposed in this study, it is anticipated that a more robust and stable learning process can be achieved, particularly in the presence of process variations.

S2. Detailed modeling of write disturbance tendencies for various HS cases

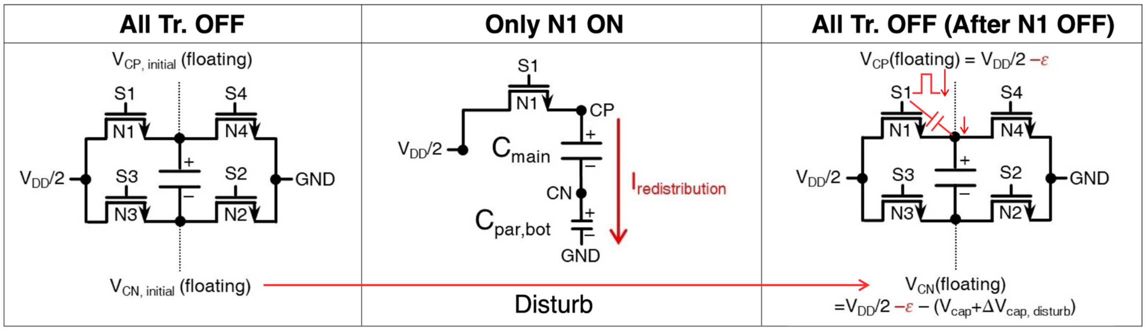

Figure S6. Write disturbance mechanism in a 6T1C cell with only the N1 transistor turned on.

(1) Derivation process of Equations (2) and (3) in the main text

In the 6T1C device, both nodes of the capacitor (CP and CN) are floating, which causes CP and CN nodes to fluctuate simultaneously even when only one transistor is selected. This fluctuation leads to charge redistribution between the main capacitor and the parasitic capacitors, resulting in a change in V_{cap}. To analyze this phenomenon mathematically, a charge redistribution model was utilized to derive an analytical model.

For instance, when the N1 transistor connected to the CP node is selected, the CP node's voltage is fixed to $V_{DD}/2$, resulting in voltage fluctuation at the CN node, causing a current to flow. This current consists of capacitive current from the main capacitor and capacitive current from parasitic capacitors connected to the CN node. By applying Kirchhoff's Current Law to these two types of currents, we derive Equation (1).

The change in V_{cap} is derived by subtracting the voltage change at the CN node from the voltage change at the CP node ($\Delta V_{cap,disturb} = \Delta V_{CP} - \Delta V_{CN}$). By extending Equation (1), the disturbance in V_{cap} caused by the N1 selection is expressed in Equation (2). This equation indicates that the disturbance is proportional to the voltage change at the CP node and the parasitic capacitance attached to the CN node due to the selection of the N1 transistor. This relationship applies similarly to scenarios where the N4 selection attached to the CP node is activated. Likewise, when analyzing the disturbance caused by the selection of the N2 or N3 transistor connected to the CN node, the results are expressed in Equation (3). From this equation, it can be observed that the disturbance caused by the selection of a transistor at the CN node is proportional to the voltage change at the CN node and the parasitic capacitance attached to the CP node.

(2) Derivation process of Equation (5) in the main text

In the case of the Disturbance Neutralization Operation (DNO), when N1 or N4 connected to the upper node in the array is selected, N1 is always turned on. Similarly, when N2 or N3 connected to the lower node is selected, N3 is always turned on, ensuring that the voltage at the CP or CN node is consistently maintained at approximately $V_{DD}/2$. Consequently, disturbances occur when the two floating nodes (CP and CN) are alternately selected. When the transistor connected to the lower node is selected first, followed by the transistor connected to the upper node, the resulting voltage change at CP (ΔV_{CP}) becomes $-V_{cap}$, leaving only an exponentially decaying disturbance as described in Equation (4). Conversely, when the transistor connected to the upper node is selected first, followed by the transistor connected to the lower node, the voltage change at CN (ΔV_{CN}) becomes V_{cap}, resulting in an exponentially decaying disturbance as described in Equation (5).

(1) $I = C_{main}\left(\frac{\partial V_{CP}}{\partial t} - \frac{\partial V_{CN}}{\partial t}\right) = C_{par,bot}\frac{\partial V_{CN}}{\partial t}$

(2) $\Delta V_{cap,disturb} = \frac{C_{par,bot}}{C_{main}+C_{par,bot}}\Delta V_{CP}$

(3) $\Delta V_{cap,disturb} = -\frac{C_{par,top}}{C_{main}+C_{par,top}}\Delta V_{CN}$

(4) $\Delta V_{cap,disturb} = -\frac{C_{par,bot}}{C_{main}+C_{par,bot}}V_{cap}$

(5) $\Delta V_{cap,disturb} = -\frac{C_{par,top}}{C_{main}+C_{par,top}}V_{cap}$

S3. Verification of 6T1C convergence under increased pulse application

Under DNO, V_{cap} must stably converge to zero even when a large number of pulses are applied, ensuring that inaccurate information (weight) is not recorded due to continuous pulses. To verify this, we applied more pulses than those presented in Figure 3 of the main text and examined the behavior of the 6T1C device in the fully converged state. The results, shown in Figure S7, demonstrate that even with a substantial number of pulses (10,000), the use of DNO ensures stable convergence without deviation from $V_{cap} = 0$.

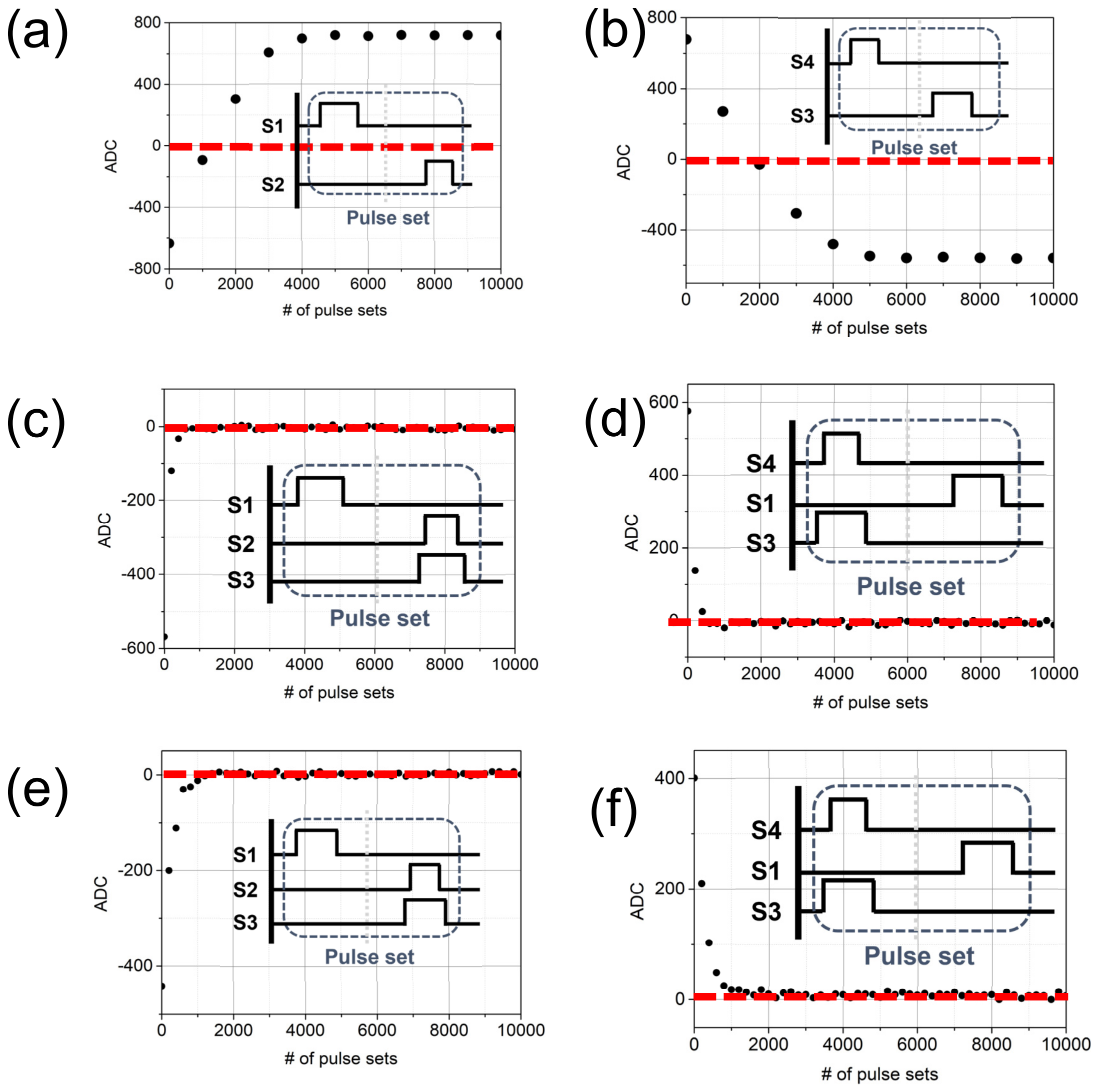

Figure S7. Measurement results of the full convergence state after applying 10,000 pulse sets. The red dashed line represents the ADC value at $V_{cap} = 0$.

S4. Results on unintentional transistor activation due to applied disturbance neutralization bias(DNB).

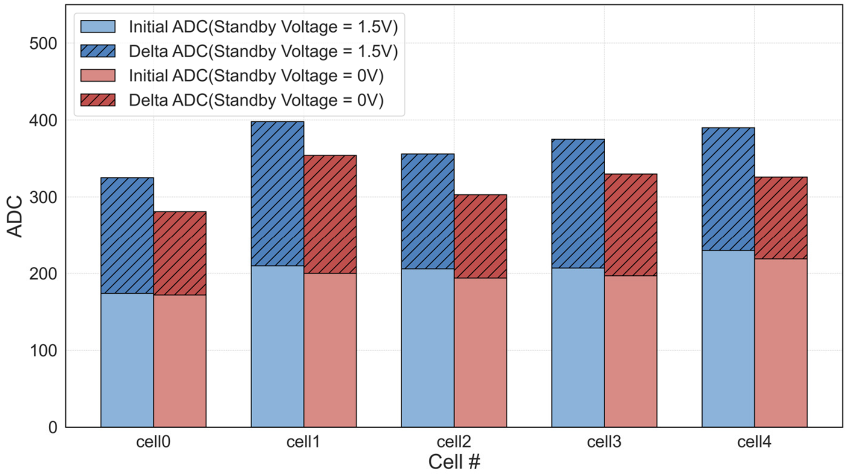

Figure S8. The measurement results from the 6T1C 5x1 array. The blue bars represent the measurements with a DNB of 1.5V applied, while the red bars show the measurements without the DNB. The hatched bars indicate the change in ADC observed after 500 repetitions of HS2 (conventional). As explained in Equations (6), (7) of the main text, applying DNB exacerbates unintentional transistor activation due to capacitive coupling effects in actual device measurement results.

S5. Die-to-die variation of an 8-inch wafer for parameter extraction in Monte Carlo Simulations.

1. Process variation

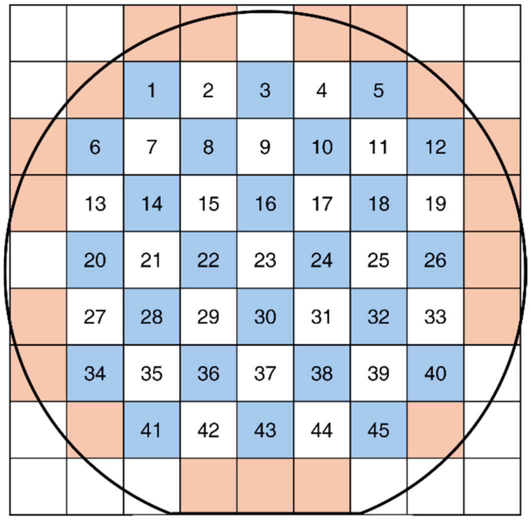

Parameters from massive measurement results of the 8-inch wafer	
Parameter	Median $\pm$ sigma
SS(mV /dec)	134.8 $\pm$ 11.1
Mobility (cm^2/VS)	3.71$\pm$ 0.60
On current (Vg = 1V)	1.1E-7 $\pm$ 4.7E-8
Vth(V)	-0.51 $\pm$ 0.18

Figure S9. The 6T1C synaptic devices and single IGZO TFTs were fabricated on an 8-inch wafer, consisting of a total of 45 dies. To extract the variation data for V_{th}, and mobility of the single IGZO TFTs, massive measurements were performed on 23 dies, highlighted in blue in the figure using Labview and auto-prober equipment. As a result, the data presented in the table was obtained, and these parameters were incorporated into the SPICE Monte Carlo simulation.

2. Voltage & temperature variation

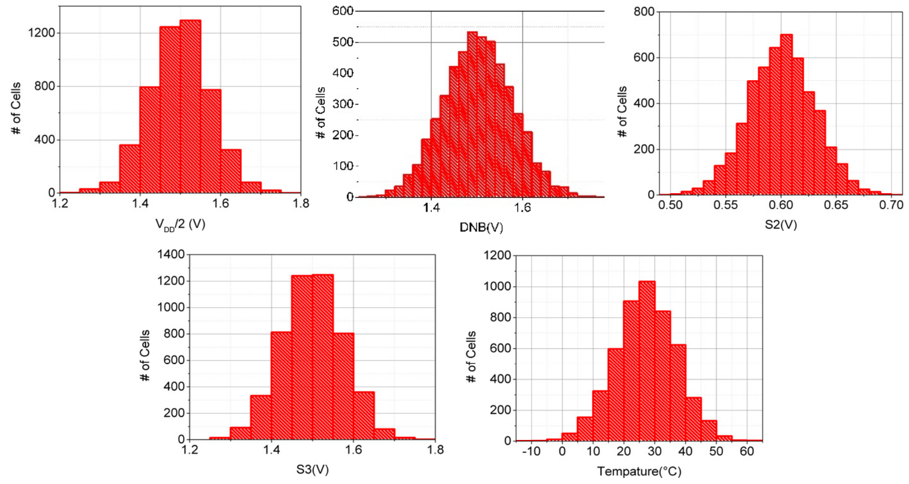

Figure S10. Voltage and temperature distributions used in Monte Carlo simulation.

S6. SPICE Monte Carlo simulation results based on initial weight and various pulse scenarios

The results of Monte Carlo simulation were obtained by incorporating the variability from the massive measurement data in S5. Variations in threshold voltage(V_{th}) and channel width were included in the simulation. Specifically, the V_{th} variability data from Figure S9 were used for V_{th}, while the mobility variation from Figure S9 was applied for channel width. The channel width introduces variation in the parasitic capacitance within the device, allowing the analysis of the half-selected issue caused by the variability in parasitic capacitance components. The figures below illustrate the changes in V_{cap} after 100 consecutive pulses were applied for different initial V_{cap} values (-1.5V, -0.75V, 0V, 0.75V, 1.5V) under different pulse scenarios. Pulses were applied consecutively for all half-selected cases, including potentiation and depression.

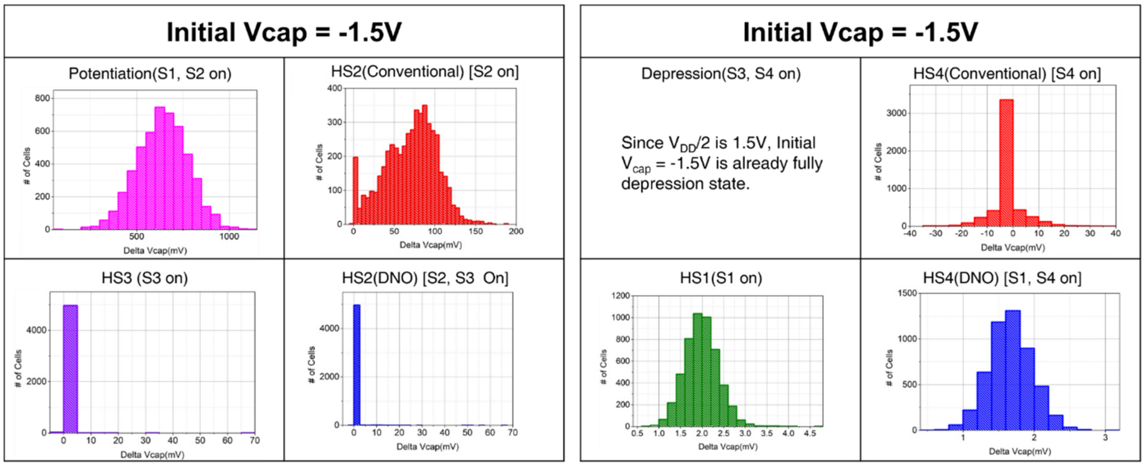

Figure S11. The results demonstrate the variation in V_{cap} after 100 consecutive pulses were applied under different pulse scenarios, with an initial V_{cap} of -1.5V. The simulation accounts for variation in V_{th}, channel width, voltage, and temperature, analyzing a total of 5,000 cells.

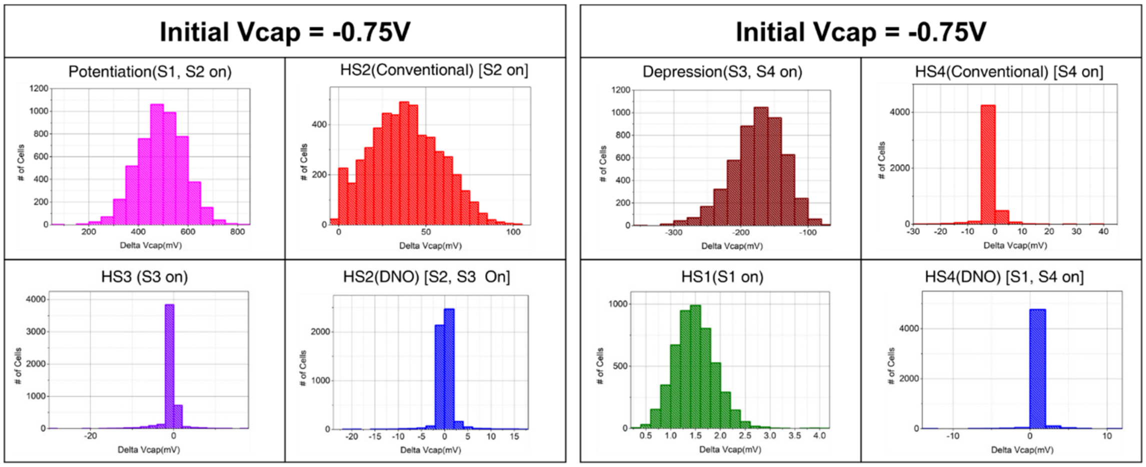

Figure S12. The results demonstrate the variation in V_{cap} after 100 consecutive pulses were applied under different pulse scenarios, with an initial V_{cap} of -0.75V. The simulation accounts for variation in V_{th}, channel width, voltage, and temperature, analyzing a total of 5,000 cells.

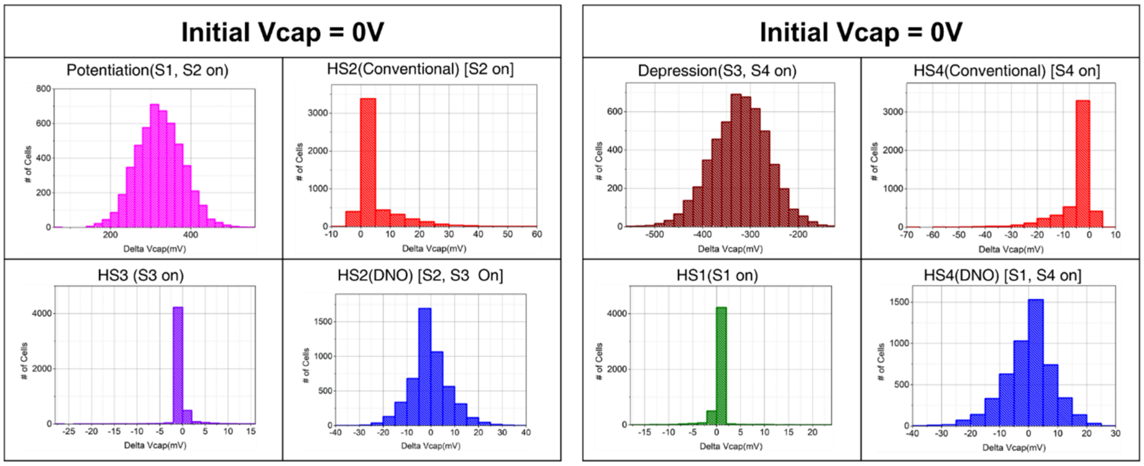

Figure S13. The results demonstrate the variation in V_{cap} after 100 consecutive pulses were applied under different pulse scenarios, with an initial V_{cap} of 0V. The simulation accounts for variation in V_{th}, channel width, voltage, and temperature, analyzing a total of 5,000 cells.

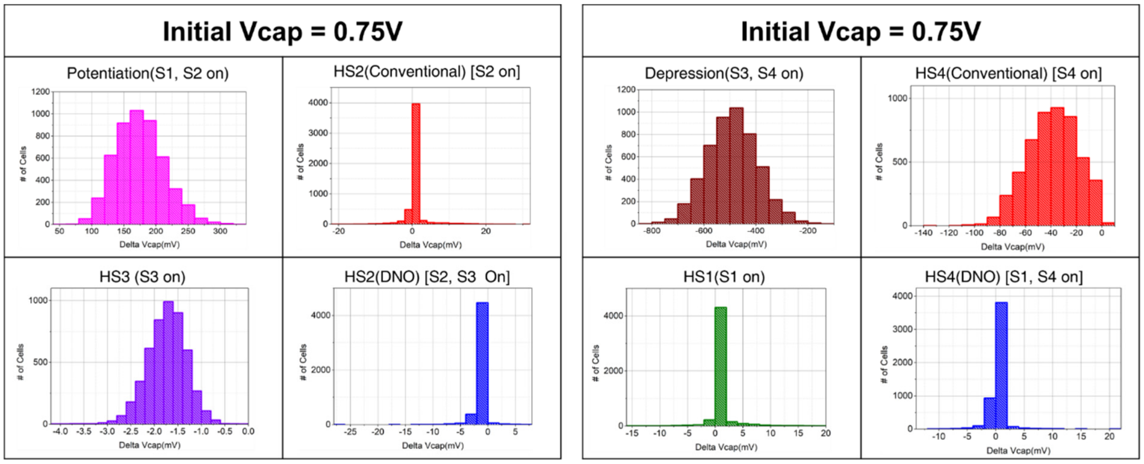

Figure S14. The results demonstrate the variation in V_{cap} after 100 consecutive pulses were applied under different pulse scenarios, with an initial V_{cap} of 0.75V. The simulation accounts for variation in V_{th}, channel width, voltage, and temperature, analyzing a total of 5,000 cells.

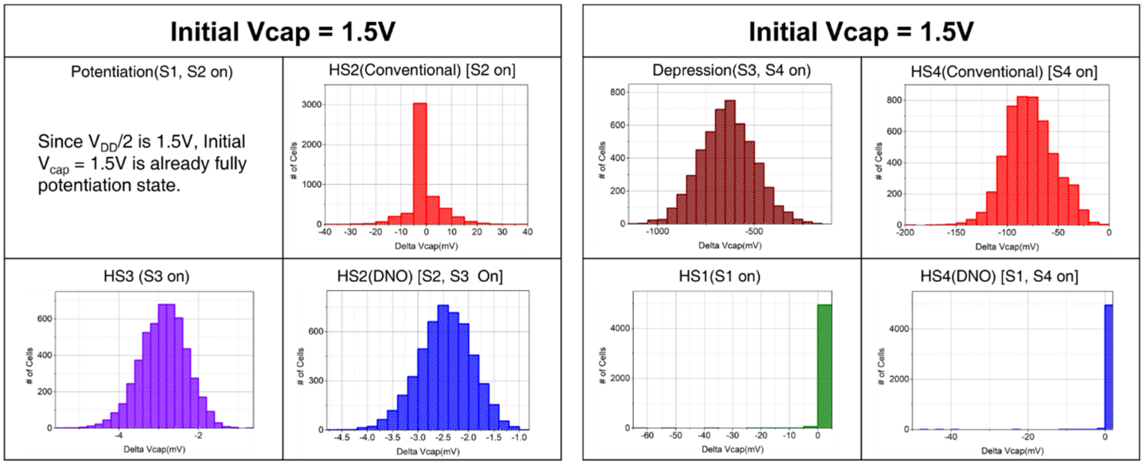

Figure S15. The results demonstrate the variation in V_{cap} after 100 consecutive pulses were applied under different pulse scenarios, with an initial V_{cap} of 1.5V. The simulation accounts for variation in V_{th}, channel width, voltage, and temperature, analyzing a total of 5,000 cells.

S7. Integration of SPICE-6T1C array DNN simulation for disturbance-aware training

(a)

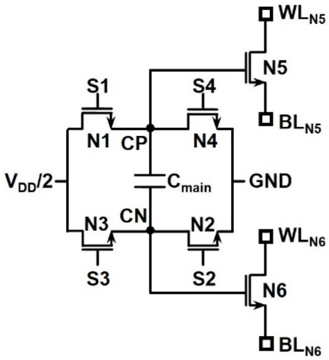

(b)

	S1 On		S1 Off	
**S2 On**	Potentiation		HS2	
	ΔV_{CP}	ΔV_{CN}	ΔV_{CP}	ΔV_{CN}
**S2 Off**	HS1		Unselected	
	ΔV_{CP}	ΔV_{CN}		

	S3 On		S3 Off	
**S4 On**	Depression		HS4	
	ΔV_{CP}	ΔV_{CN}	ΔV_{CP}	ΔV_{CN}
**S4 Off**	HS3		Unselected	
	ΔV_{CP}	ΔV_{CN}		

	WL_{N5} On	
**S3 On**	N5 read	
	ΔV_{CP}	ΔV_{CN}

	WL_{N6} On	
**S1 On**	N6 read	
	ΔV_{CP}	ΔV_{CN}

(c)

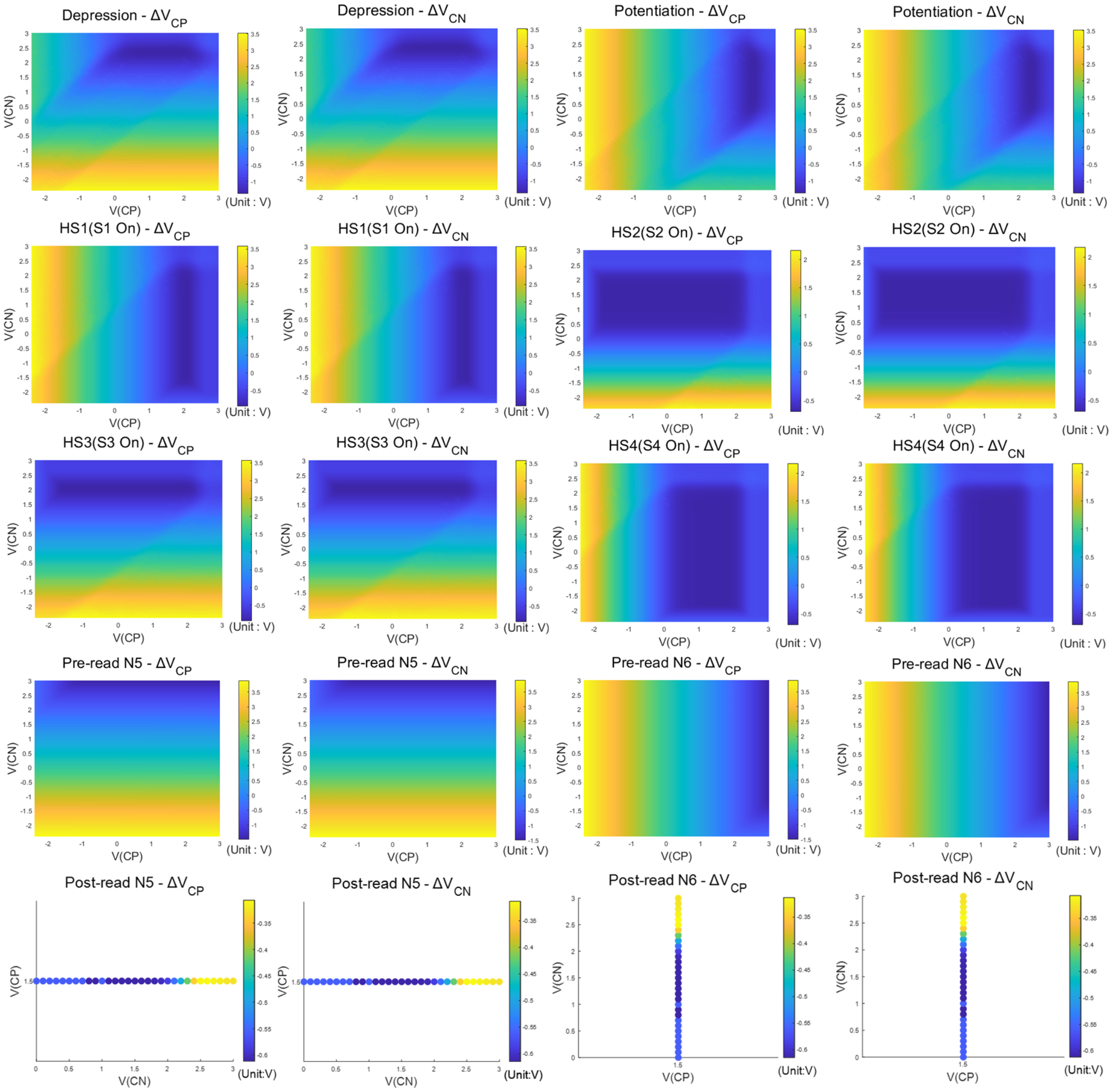

Figure S16. (a) Schematic of the 6T1C device (b) Transistor selection scenarios for a 6T1C cell under conventional operation (c) Color map showing the voltage changes at the CP and CN nodes after the application of a single pulse according to the scenarios in (b). Post-read N5 and Post-read N6 illustrate the change of the CP and CN voltages following the completion of the read operation, as the read pulse is turned off. During the N5 and N6 read operations, the CN and CP nodes are both held constant at $V_{DD}/2$(1.5V). Accordingly, the voltage change was extracted under the condition where the initial voltages of both nodes were set to 1.5V.

As shown in Figure S16-(a), all nodes of the 6T1C cell, except for the CP and CN nodes (V_{DD}/2, S1, S2, S3, S4, WLN5, BLN5, WLN6, BLN6, GND), are clearly defined by external voltages. The only undefined voltages in the 6T1C cell are those of the CP and CN nodes. Therefore, by recording ΔV_{CP} and ΔV_{CN} for the arbitrary transistor selection scenarios summarized in Figure S16-(b), it is possible to track the cell's next state after any pulse is applied in any given situation. The color map in Figure S16-(c) illustrates the voltage changes at the CP and CN nodes following a single pulse application. All color maps in (c) were extracted through HSPICE simulations, with parasitic components of the IGZO TFT set based on values published in a paper that accurately measured these components.[10,11] The V_{DD}/2 of the 6T1C cell was set to 1.5V for this experiment, so the maximum possible values for V_{CP} and V_{CN} are 3V, while the minimum values, allowing for the capacitive coupling effect and associated voltage drops, were conservatively set to -2.4V. Using the lookup table in Figure S16-(c), a 6T1C software simulation was conducted, enabling disturbance-aware training simulations that take real-time write disturbances caused by HS cases into account. When the DNO is applied, as shown in Figure S17-(a), the pulse scenarios for the 6T1C cell change and the HS2(S2 on) and HS4(S4 on) color maps are replaced with the HS2(S2, S3 on) and HS4(S1, S4 on) scenarios for the simulations.

(a)

	**S1 On**		**S1 Off(S3 On)**	
**S2 On**	Potentiation		HS2	
	ΔV_{CP}	ΔV_{CN}	ΔV_{CP}	ΔV_{CN}
**S2 Off**	HS1		HS3	
	ΔV_{CP}	ΔV_{CN}	ΔV_{CP}	ΔV_{CN}

	**WL_{N5} On**	
**S3 On**	N5 read	
	ΔV_{CP}	ΔV_{CN}

	**S3 On**		**S3 Off(S1 On)**	
**S4 On**	Depression		HS4	
	ΔV_{CP}	ΔV_{CN}	ΔV_{CP}	ΔV_{CN}
**S4 Off**	HS3		HS1	
	ΔV_{CP}	ΔV_{CN}	ΔV_{CP}	ΔV_{CN}

	**WL_{N6} On**	
**S1 On**	N6 read	
	ΔV_{CP}	ΔV_{CN}

(b)

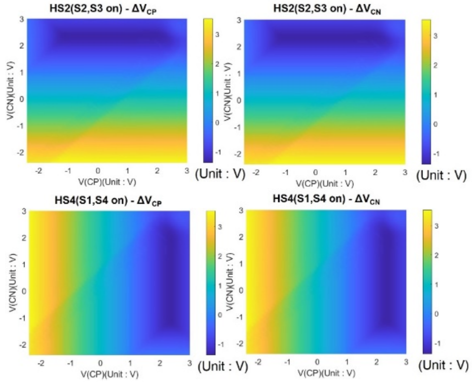

Figure S17. (a) Transistor selection scenarios for a 6T1C cell under DNO described in 2.1 Section in the main text (b) According to the DNO, the HS2(S2 on) and HS4(S4 on) cases are no longer present, and are replaced by newly introduced scenarios, HS2(S2, S3 on) and HS4(S1, S4 on), which share the same names but involve different selected transistors. Consequently, the HS2(S2 on) and HS4(S4 on) color maps in Fig. S16-(c) are replaced with the HS2(S2, S3 on) and HS4(S1, S4 on) color maps.

S8. The difference in the degree of improvement between the fully connected layer and the convolutional layer due to the application of the disturbance neutralization operation and the bias.

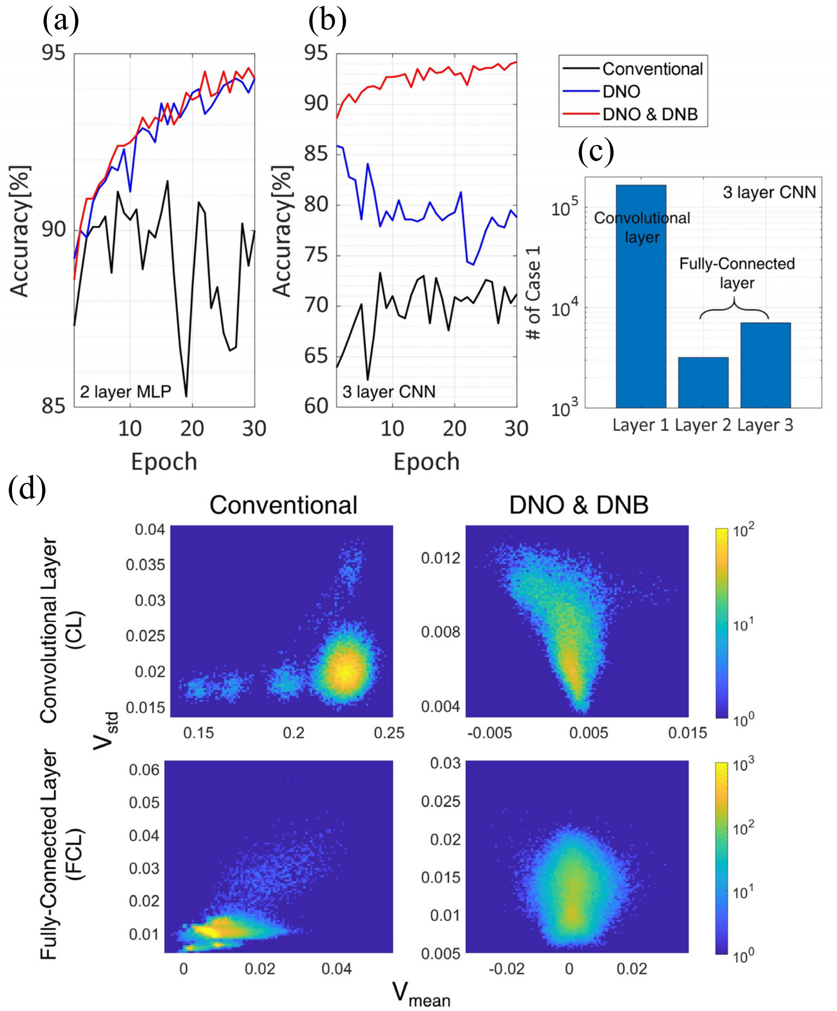

Figure S18. Effects of disturbance neutralization operation and disturbance neutralization bias. (a) Accuracy improvement in a Multi-Layer Perceptron (MLP) consisting of two layer fully connected layers (FCL). (b) Accuracy improvement in a Convolutional Neural Network (CNN) consisting of one convolutional layer and two layer FCLs. (c) Number of case 1 occurrences during training in each layer of the CNN mentioned in (b). (d) Change in V_{cap} distribution due to disturbance neutralization operation and disturbance neutralization bias of the convolutional layer (CL) and FCL in the CNN mentioned in (b).

S9.Pseudo code of Pulse Scheduling (PS)

```
Algorithm: Pulse Scheduling (PS)

PROCEDURE PS(CA, I, G) // CA: Crossbar Array, I: Input, G: Gradient
        PULSE_P=ZEROS(M,J), PULSE_D=ZEROS(M,J), PULSE_I=ZEROS(KxKyC, J) // J: total number of update
        FOR j= 0 TO J-1
                FOR u = 0 TO M-1
                        flag_gradient = RANDOM(0, 1)
                        IF flag_gradient < α|G[u,j]| THEN
                                // α: Coefficient considering parameters such as learning rate..
                                IF G[u,j] <0 THEN
                                        PULSE_P[u,j] = 1
                                        //gradient pulse for potentiation
                                ELSE
                                        PULSE_D[u,j] = 1
                                        // gradient pulse for depression
                                END IF
                        END IF
                END FOR
                FOR u = 0 TO KxKyC-1
                        flag_input = RANDOM(0, 1)
                        IF flag_input < βI[u,j] THEN
                                // β: Coefficient considering parameters such as learning rate..
                                PULSE_I[u]=1        // input pulse for update
                        END IF
                END FOR
        END FOR
        FOR j=0 TO J-1
                POTENTIAION(CA, PULSE_I[:,j], PULSE_P[:,j])
        END FOR
        FOR j=0 TO J-1
                DEPRESSION(CA, PULSE_I[:,j], PULSE_D[:,j])
        END FOR
END PROCEDURE
```

Figure S19. Pseudo code of Pulse Scheduling. All gradients for updating the convolutional kernel weights are applied by completing the potentiation phase first, followed by the depression phase.

S10. Development of an energy lookup table for calculating energy consumption during the training process

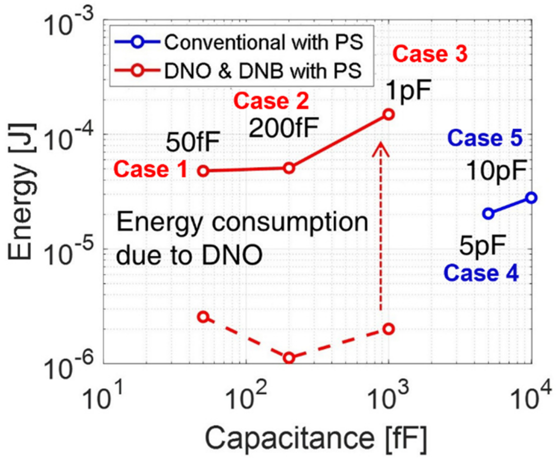

Figure S20. Total energy consumed for learning across various capacitor sizes and learning methods (cases 1-5). The total energy values were obtained using the table shown below.

As detailed in Section S7, the 6T1C device can track its response to arbitrary pulses by recording changes in the CP and CN nodes. This scheme allows for the precise calculation of the energy consumed during the training process. The current through N1, N2, N3, and N4 is determined by the voltage changes at the CP and CN nodes for any given transistor selection scenario (Potentiation, Depression, HS1, HS2(conventional), HS2(DNO), HS3, HS4(conventional), HS4(DNO)). Additionally, the real-time resistance of the N1, N2, N3, and N4 transistors is also defined by the voltage changes of the CP and CN nodes. Through SPICE, we can track these values in real time, recording the time-dependent current and resistance during the selection scenarios. By integrating the I^2R values over time, we generated a look-up table that records the total energy consumed by the N1-N4 transistors during a single pulse application, which varies according to the initial voltage at the CP and CN nodes. The table below shows the total energy consumption for each transistor selection scenario in the 6T1C circuit when a single pulse is applied across cases 1 to 5. This table allows for the accurate calculation of total energy consumption across various scenarios until training is complete, as shown in Figure S20.

Case 1 – Cell capacitance: 50fF
Training method : DNO & DNB(1.5V)

Depression(S3, S4 on) – Energy

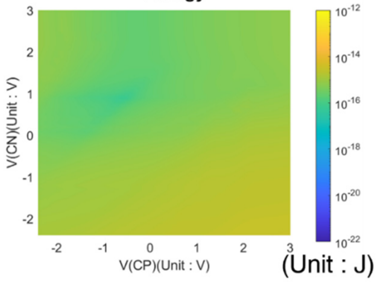

Potentiation(S1, S2 on) – Energy

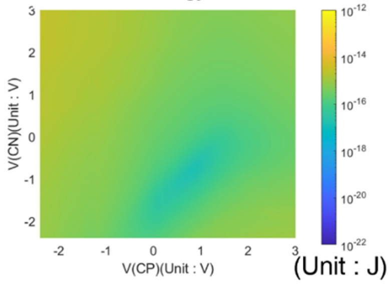

HS1(S1 on) – Energy

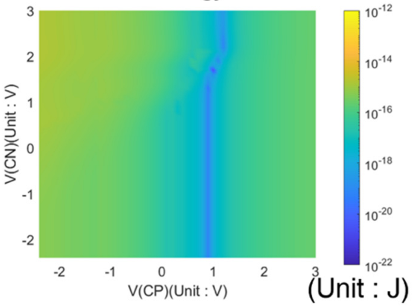

HS2(Conventional) [S2 on] – Energy

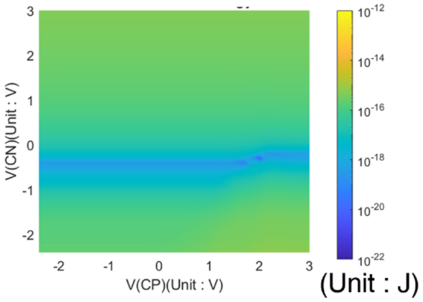

HS3(S3 on) – Energy

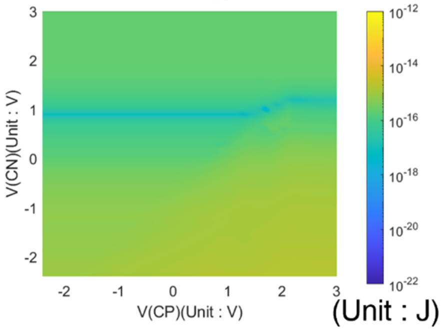

HS4(Conventional) [S4 on] – Energy

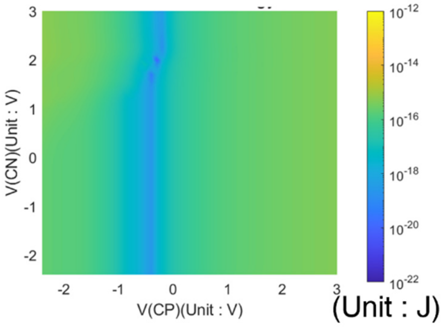

HS2(DNO)[S2, S3 on] – Energy

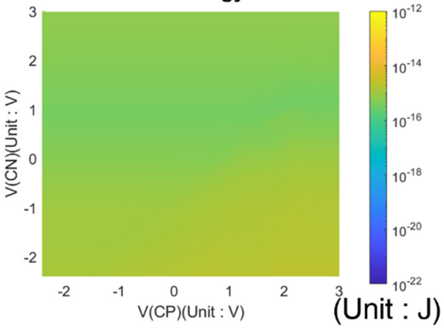

HS4(DNO) [S1,S4 on] – Energy

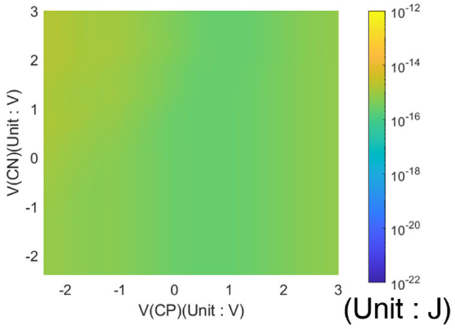

Case 2 – Cell capacitance: 200fF
Training method : DNO & DNB(1.5V)

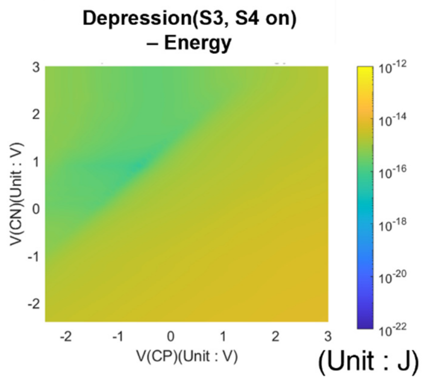

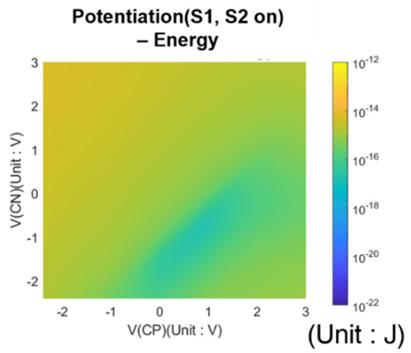

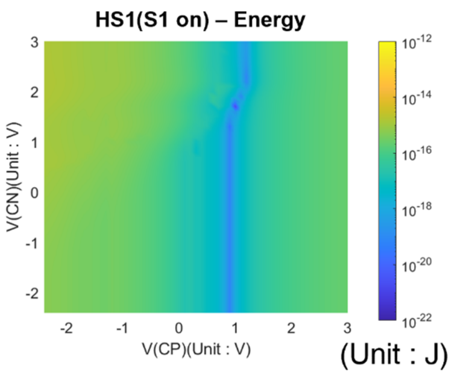

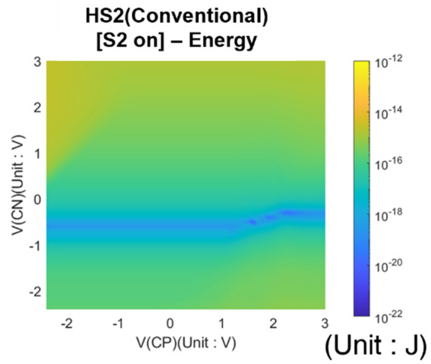

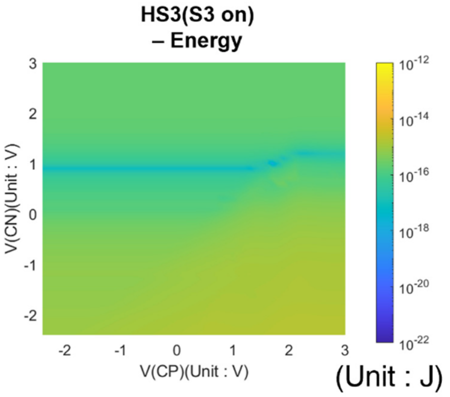

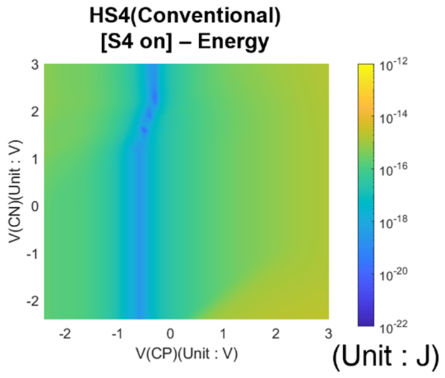

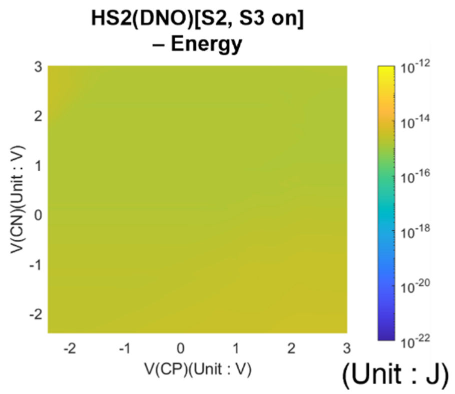

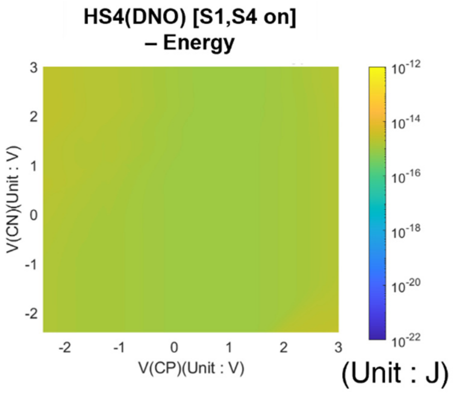

Case 3 – Cell capacitance: 1pF
Training method : DNO & DNB(1.5V)

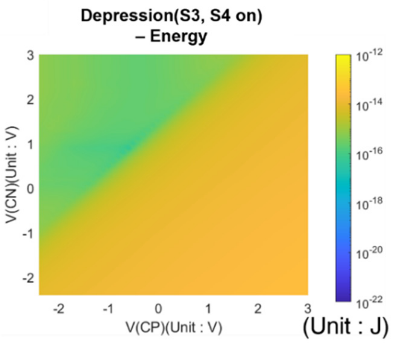

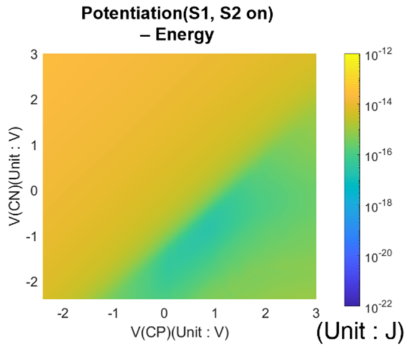

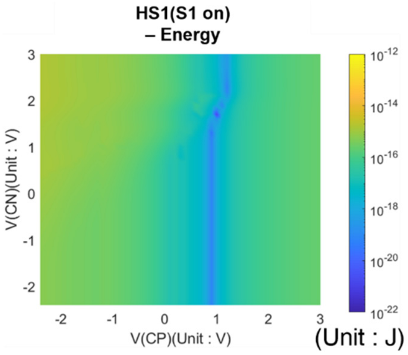

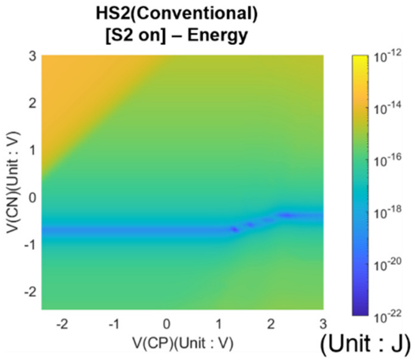

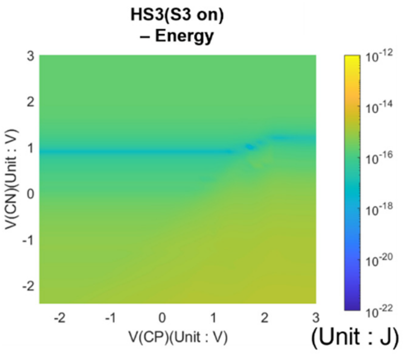

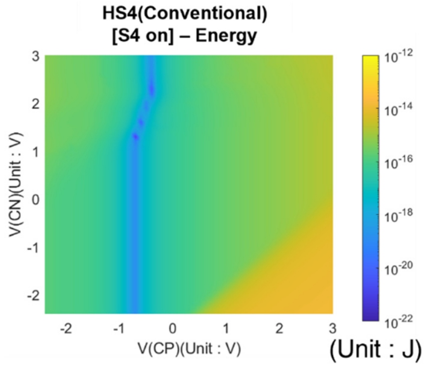

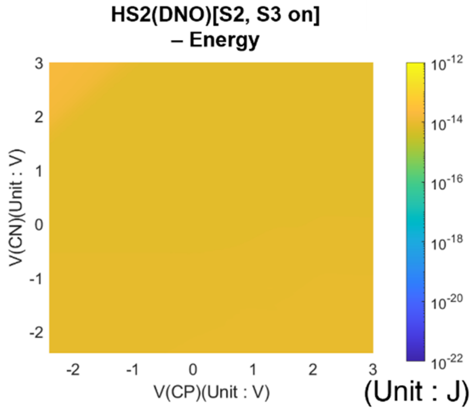

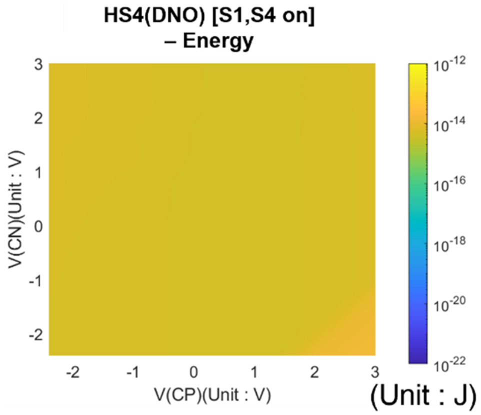

Case 4 – Cell capacitance: 5pF
Training method: Conventional operation

Potentiation(S1, S2 on)
– Energy

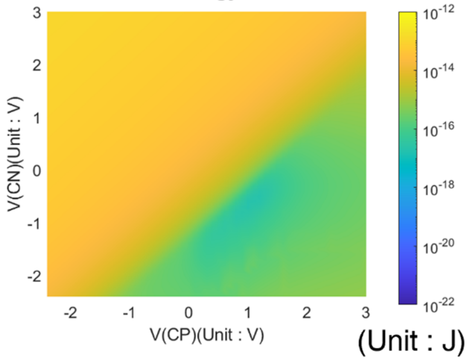

Depression(S3, S4 on)
– Energy

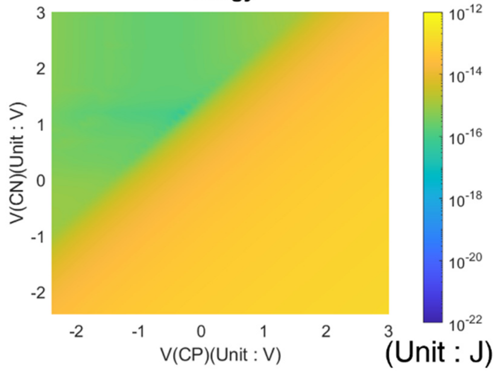

HS1(S1 on)
– Energy

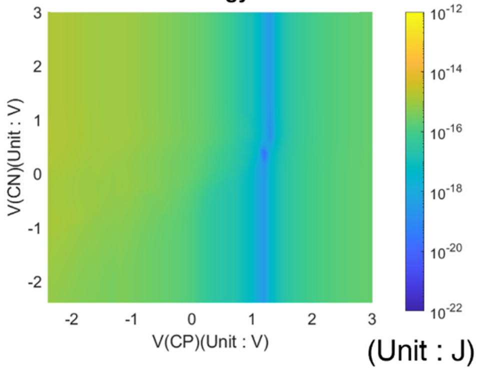

HS2(Conventional)
[S2 on] – Energy

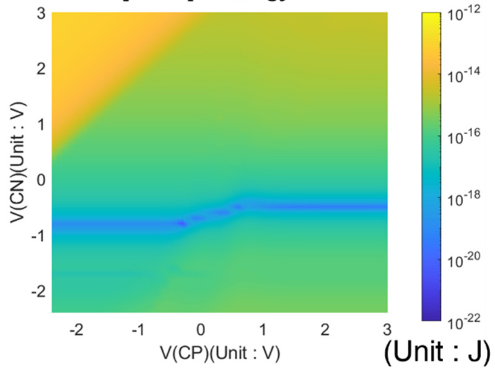

HS3(S3 on)
– Energy

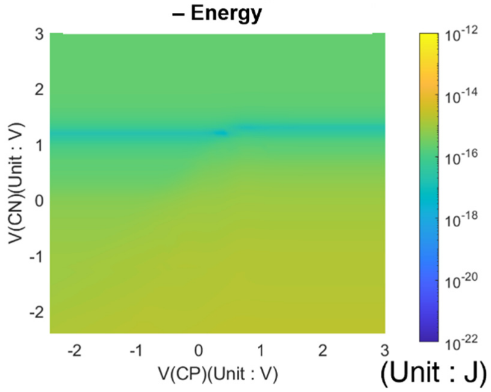

HS4(Conventional)
[S4 on] – Energy

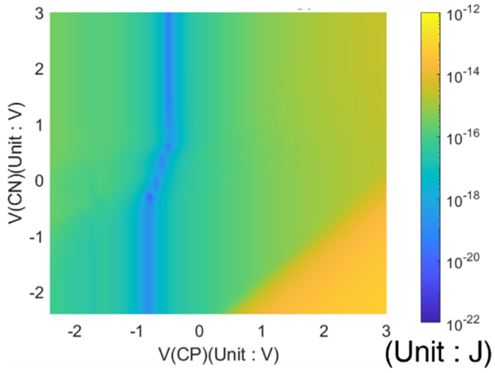

Case 5 – Cell capacitance: 10pF
Training method: Conventional operation

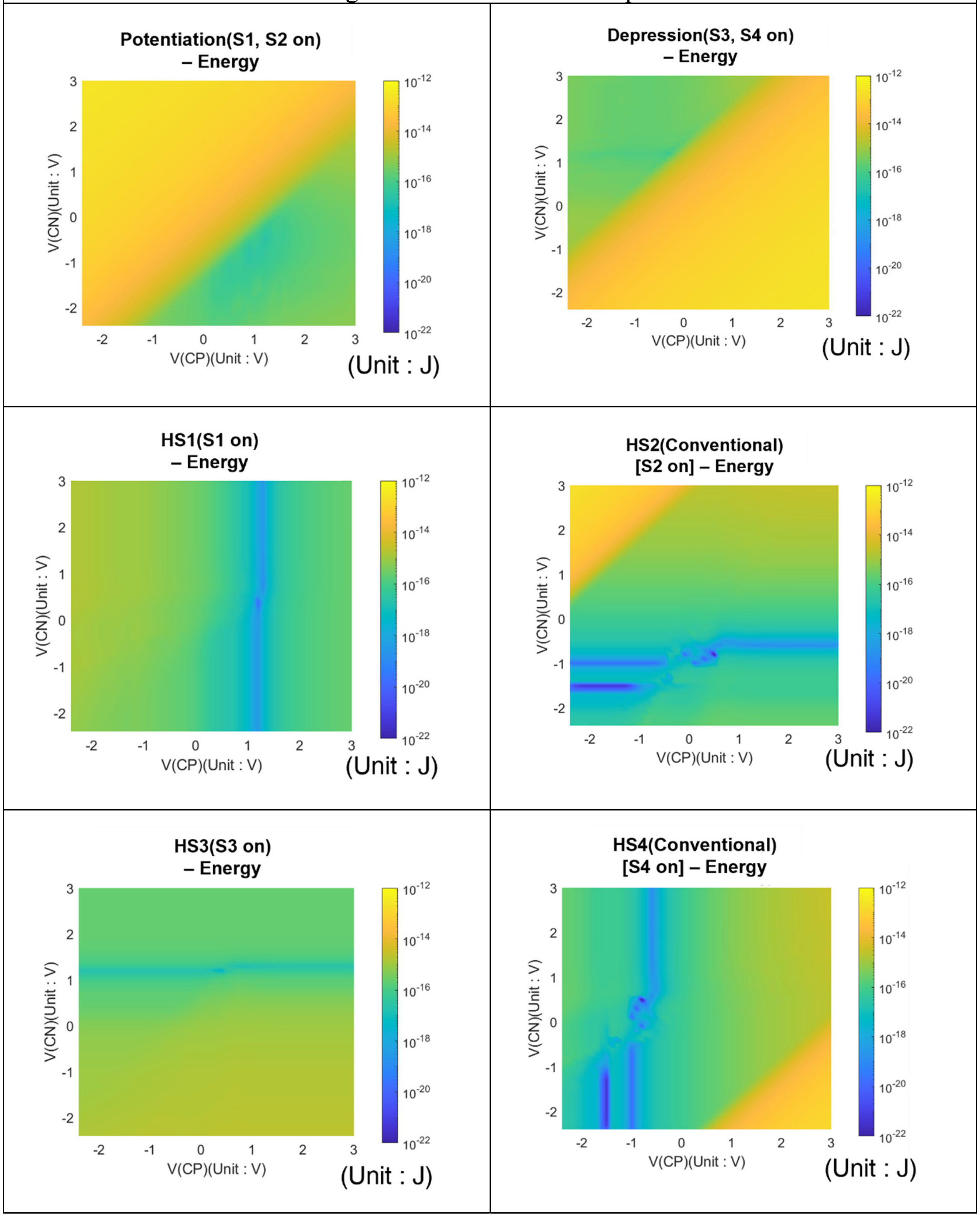

Figure S21. Energy look-up tables used for each case to generate Figure S20.

Reference

[1] T. Gokmen, W. Haensch, *Front. Neurosci.* **2020**, *14*, 1.

[2] T. Gokmen, *Front. Artif. Intell.* **2021**, *4*, 699148.

[3] C. Lee, K. Noh, W. Ji, T. Gokmen, S. Kim, *Front. Neurosci.* **2022**, *15*, 1.

[4] M. J. Rasch, F. Carta, O. Fagbohungbe, T. Gokmen, *Nat. Commun.* **2024**, *15*, DOI 10.1038/s41467-024-51221-z.

[5] M. Liu, Z. Li, W. Lu, K. Chen, J. Niu, F. Liao, Z. Wu, C. Lu, W. Z. Li, D. Geng, N. Lu, C. Dou, G. Yang, L. Li, M. Liu, *Dig. Tech. Pap. - Symp. VLSI Technol.* **2024**, 2.

[6] H. Baba, S. Ohshita, T. Hamada, Y. Ando, R. Hodo, T. Ono, T. Hirose, Y. Kurokawa, T. Murakawa, H. Kunitake, T. Nakura, M. Kobayashi, H. Yoshida, M. C. Chen, M. H. Liao, S. Z. Chang, S. Yamazaki, *Tech. Dig. - Int. Electron Devices Meet. IEDM* **2021**, *2021-Decem*, 21.2.1.

[7] J. Won, J. Kang, S. Hong, N. Han, M. Kang, Y. Park, Y. Roh, H. J. Seo,C. Joe, U. Cho, M. Kang, M. Um, K.-H. Lee, J.-E. Yang, M. Jung, H.-M.Lee, S. Oh, S. Kim, S. Kim, *Adv. Sci.* ***2023**, 10, 29.*

[8] A. Grossi, E. Vianello, M. M. Sabry, M. Barlas, L. Grenouillet,J. Coignus, E. Beigne, T. Wu, B. Q. Le, M. K. Wootters, C.Zambelli, E. Nowak, S. Mitra, *IEEE Trans. Electron Devices* **2019**, *66,1281.*

[9] A. Grossi, E. Nowak, C. Zambelli, C. Pellissier, S. Bernasconi, G.Cibrario, K. El Hajjam, R. Crochemore, J. F. Nodin, P. Olivo, L.Perniola,presen`ted at *Int. Electron Devices Meeting, San Francisco,CA, USA* **2016**

[10] Y. Sekine, K. Furutani, Y. Shionoiri, K. Kato, J. Koyama, S. Yamazaki, *ECS Trans*. **2019**, *37, 77.*

[11] K. Kato, Y. Shionoiri, Y. Sekine, K. Furutani, T. Hatano, T. Aoki, M. Sasaki, H. Tomatsu, J. Koyama, S. Yamazaki, *Jpn. J. Appl. Phys*. **2012**, *51, 021201.*
